# A multiscale approach to understanding the shared blue-orange flower color polymorphism in two *Lysimachia* species

**DOI:** 10.1186/s12870-024-05481-y

**Published:** 2024-09-30

**Authors:** Mercedes Sánchez-Cabrera, Eduardo Narbona, Montserrat Arista, Pedro L. Ortiz, Francisco J. Jiménez-López, Amelia Fuller, Benjamin Carter, Justen B. Whittall

**Affiliations:** 1 Departmento de Biología Vegetal y Ecología, Facultad de Biología, Universidad de Sevilla, Sevilla, 41012 España; 2 Departamento de Biología Molecular e Ingeniería Bioquímica, Universidad Pablo de Olavide, Sevilla, 41013 España; 3 Departamento de Biología y Geología, Física y Química Inorgánica, Universidad Rey Juan Carlos (URJC), Móstoles, 28933 España; 4 Instituto de Investigación en Cambio Global (IICG-URJC), Universidad Rey Juan Carlos, Móstoles, 28933 España; 5 Department of Chemistry and Biochemistry, Santa Clara University, Santa Clara, CA 95053 USA; 6 Department of Biological Sciences, San Jose State University, San Jose, CA 95182 USA; 7 Department of Biology, Santa Clara University, Santa Clara, CA 95053 USA

**Keywords:** Flower color polymorphism, Anthocyanin, Flavonoid, *Lysimachia arvensis*, *Lysimachia monelli*, Petal transcriptome

## Abstract

**Background:**

Polymorphisms are common in nature, but they are rarely shared among closely related species. Polymorphisms could originate through convergence, ancestral polymorphism, or introgression. Although shared neutral genomic variation across species is commonplace, few examples of shared functional traits exist. The blue-orange petal color polymorphisms in two closely related species, *Lysimachia monelli* and *L. arvensis* were investigated with UV-vis reflectance spectra, flavonoid biochemistry, and transcriptome comparisons followed by climate niche analysis.

**Results:**

Similar color morphs between species have nearly identical reflectance spectra, flavonoid biochemistry, and ABP gene expression patterns. Transcriptome comparisons reveal two orange-specific genes directly involved in both blue-orange color polymorphisms: *DFR-2* specificity redirects flux from the malvidin to the pelargonidin while *BZ1-2* stabilizes the pelargonidin with glucose, producing the orange pelargonidin 3-glucoside. Moreover, a reduction of *F3’5’H* expression in orange petals also favors pelargonidin production. The climate niches for each color morph are the same between the two species for three temperature characteristics but differ for four precipitation variables.

**Conclusions:**

The similarities in reflectance spectra, biochemistry, and ABP genes suggest that a single shift from blue-to-orange shared by both lineages is the most plausible explanation. Our evidence suggests that this persistent flower color polymorphism may represent an ancestrally polymorphic trait that has transcended speciation, yet future analyses are necessary to confidently reject the alternative hypotheses.

**Supplementary Information:**

The online version contains supplementary material available at 10.1186/s12870-024-05481-y.

## Introduction

Polymorphisms, or variation among individuals of the same species, provide rare windows into the process of adaptation and speciation [1–4]. Polymorphisms among individuals in a population or among populations of a single species, are omnipresent across the tree of life [5–7] and can be maintained by a diversity of forces including negative frequency-dependent selection [8], heterozygote advantage [9], genetic drift [10, 11], gene flow [3] and spatially or temporally variable selection [12]. Some polymorphisms are phylogenetically dispersed (e.g. bird plumage color or flower color [13]), while others reoccur in very closely related species (e.g. heterostyly in *Primula*, shell chirality in *Amphidromus*, cryptic body color in *Timema*, wing patterning in *Heliconius* [reviewed in 14], and anther-color polymorphism in *Erythronium* lilies [7]). The molecular underpinnings, persistence dynamics and evolutionary forces acting on these rare cases of shared variation are largely unexplored.

Some studies have proposed that polymorphisms facilitate the use of a wide range of environmental resources and may act as a precursor to speciation which then become fixed after divergence [6, 13, 15]. However, polymorphisms that persist across species [14] represent evolutionary enigmas. How can polymorphisms transcend species boundaries? Convergent evolution [16, 17] including developmental convergence (i.e. independent changes in gene expression), introgression [18, 19], and the maintenance of an ancestral polymorphism [2, 20] are three plausible evolutionary avenues leading to shared polymorphisms among closely related species.

Color polymorphisms are widespread in nature but only a few trans-specific cases have been reported, and mostly restricted to animals [3, 5, 6, 21]. Few examples have been investigated in plants. The closest example for flower color are the repeated transitions from blue to red flowers [22] associated with shifts from bee- to hummingbird-pollination [23–25] across a diversity of angiosperm lineages. However, in this case intraspecfic blue-red flower color polymorphisms (variation within populations) are very rare since either (1) multiple mutations are required to accomplish this shift and/or (2) the color is associated with a pollinator shift leading to reproductive isolation between the color types thereby fixing the color differences in newly formed species.

**Fig. 1:**
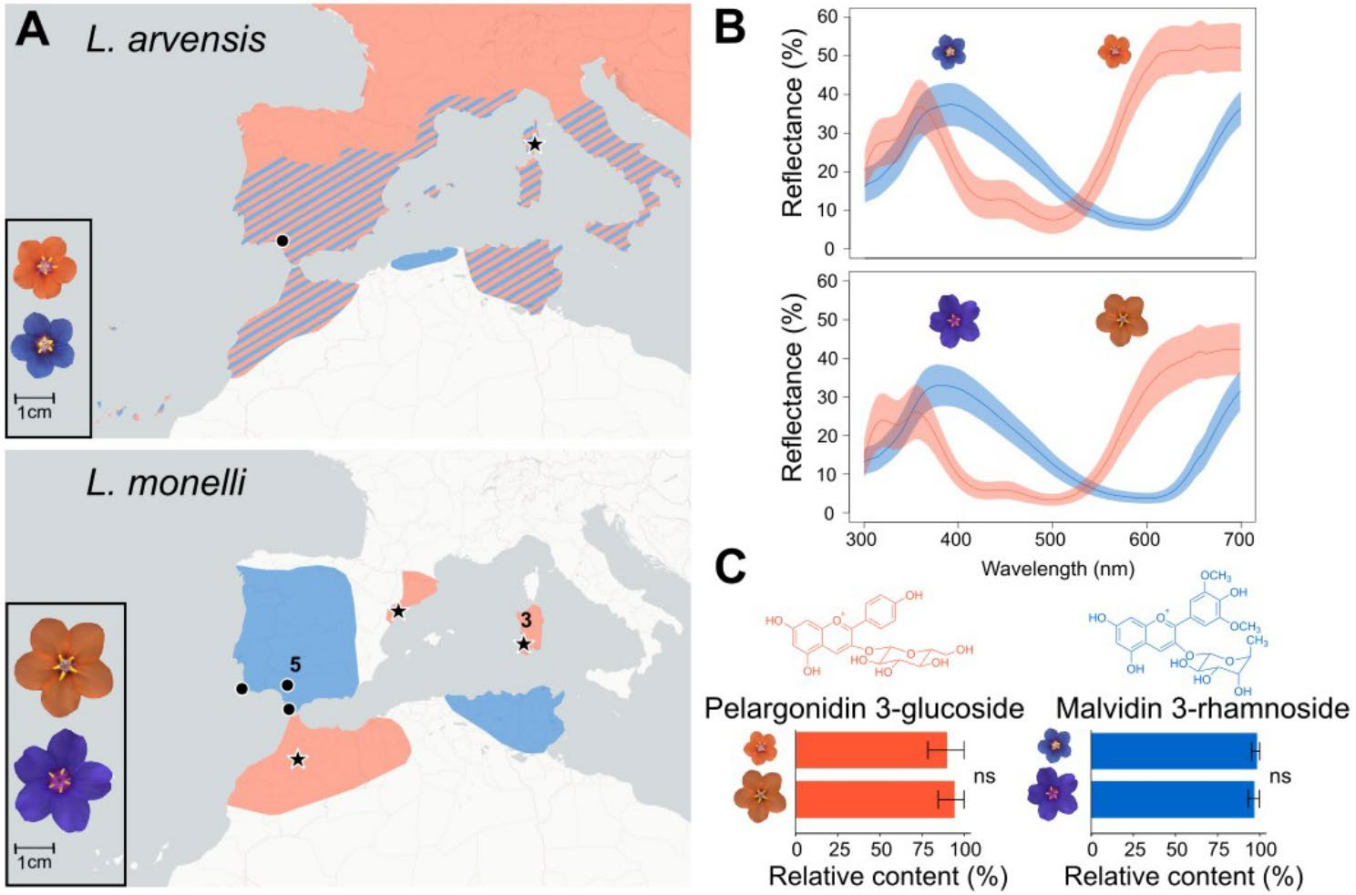
Flower color characterization of blue and orange *Lysimachia arvensis* (smaller flowers) and *L. monelli* (larger flowers). (**A**) Scaled images and geographic distributions of the two flower color morphs of both species. Coexistence of the *L. arvensis* color morphs is indicated with diagonal striping. Populations sampled for transcriptome comparisons are indicated with black circles (blue) and black stars (orange) (numbers indicate > 1 geographically adjacent populations were sampled for *L. monelli*). (**B**) UV-vis reflectance spectra of blue and orange petals of both species showing mean curves with 95% confidence intervals in colored shading above and below the mean curve. (**C**) Molecular structures and relative content from the biochemical analysis of the primary anthocyanins in both species. Non-significant differences (ns) in relative content are indicated using a Mann-Whitney U test. In *L. arvensis*, orange samples include pelargonidin 3-glucoside derivatives. Error bars represent standard error (SEM) and the upper bounds were clamped when > 100% to reflect a more biologically reasonable representation of the variability

Unlike the blue to red flower color transitions leading to speciation, the blue-orange flower color polymorphism in two closely related species, *Lysimachia arvensis* and *L. monelli* [26] (*La* and *Lm* herein, Fig. 1A), does not confer a pollinator shift [27, 28]. Instead, in *La* the color polymorphism is driven by abiotic non-pollinator agents of selection such as drought and sunlight intensity [29, 30] and temperature [31] where blue flowered individuals are fitter than orange ones in drier, sunnier, and hotter environments. *La*, is an annual native to the Mediterranean Basin and central and northern Europe. A recent autopolyploid origin has been suggested for *La* for three reasons: (1) due to the lack of ITS polymorphisms [32], (2) the presence of four identical copies of one set of chromosomes [33], (3) and the phylogenetic configuration of the group based on ITS and cpDNA sequences, which suggests *L. arvensis* and *L. monelli* are not reciprocally monophyletic (instead orange morphs are monophyletic [32]). Color morph frequencies range from 0 to 100% with a steep cline in central-southern Mediterranean region where mixed populations are common (e.g., Portugal, Spain, Italy and France [29]). The cline and resulting association with abiotic forces strongly argue against pollinator mediated selection and instead point to abiotic factors which have been substantiated in a greenhouse study [29]. In *La*, the shift from blue to orange correlates with a biochemical transition from malvidin 3-rhamnoside to pelargonidin 3-glucoside [34, 35]. Orange flowers of *La* have increased expression of *DFR-2*, a duplicate gene only found in orange-flowered individuals with non-synonymous SNPs suggesting substrate specificity for dihydrokaempferol drawing flux down the pelargonidin branch of the anthocyanin biosynthetic pathway (ABP) [35]. In contrast, *Lm* is a diploid, perennial plant with no sympatric populations. Blue flowered populations of *Lm* are more common in drier habitats of central and southwestern Iberian Peninsula and eastern North Africa; while orange-flowered populations are more often found in wetter habitats of northeastern Iberian Peninsula, western North Africa, and Sardinia [28, 36]. However, co-occurs with *La* in some populations (Fig. 1A).

Herein, we investigate the shared blue-orange flower color polymorphism in *La* and *Lm*. To compare the flower color polymorphism between species we analyze the reflectance spectra, the flavonoid biochemistry, and the color genes through transcriptomics of the petals. Finally, we discuss the origin of this shared blue-orange flower color variation in this species of *Lysimachia*.

## Results

### Petal UV-vis spectra

Reflectance spectra of petals for both species are very similar across the UV and visible wavelengths when comparing the same color morphs, yet quite different between color morphs (Fig. 1B, Supplementary Fig. S1). Spectra distances between color morphs are ~ 7x larger than spectra distances within morphs between species (Permutational Manova, R2 = 0.75 vs. 0.15; Supplementary Fig. S1). Orange petals have double UV reflectance peaks that are absent in blue petals and differ in their primary inflection points (380, 570 nm for orange and 480, 660 nm for blue).

### Petal biochemical comparisons

Biochemical analyses of petal extracts revealed the underlying differences between blue and orange morphs are nearly identical between the two species. Blue petals of both species are composed of malvidin 3-rhamnoside, however small amounts of the aglycones, malvidin and delphinidin, and a flavonol derivative were also detected in both species [35] (Fig. 1C, Supplementary Table S1). In contrast, orange petals of both species accumulate principally pelargonidin 3-glucoside and to a lesser degree, other pelargonidin derivatives [35] (Supplementary Table S1). There were no significant differences in the relative content of malvidin 3-rhamnoside and pelargonidin 3-glucoside derivatives when comparing the blue and orange samples of these two species, respectively (Fig. 1C).

### Transcriptome comparisons

#### Differential expression

**Fig. 2:**
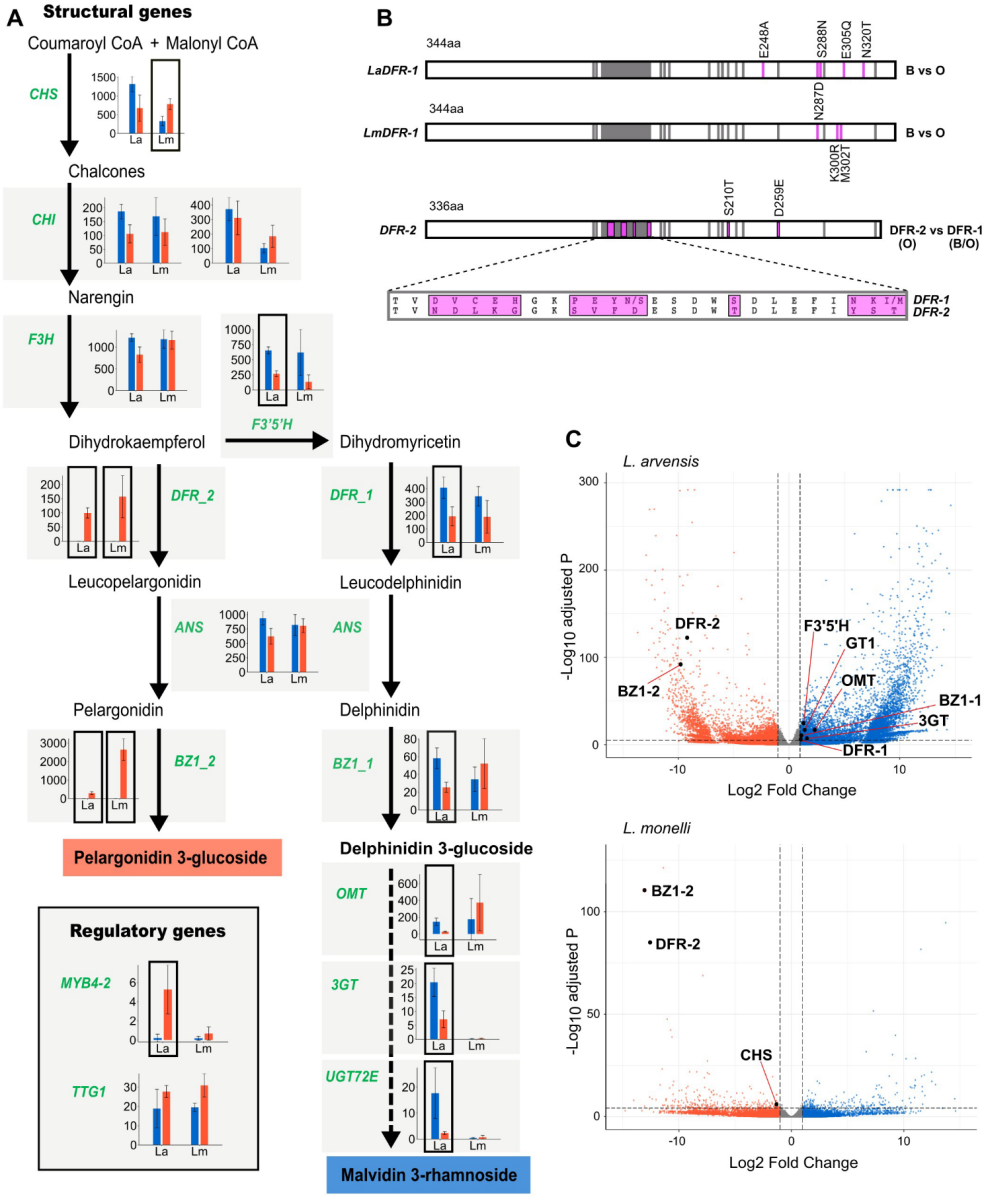
Gene expression comparisons of blue and orange morphs of two species of *Lysimachia*. (**A**) Suggested Anthocyanin Biosynthetic Pathway (ABP) producing blue (malvidin 3-rhamnoside) and orange (pelargonidin 3-glucoside) flower colors in these two species. Mean expression (TMM +/- standard error) in both species (*La* and *Lm*) are shown for each ABP gene (green font). Significantly differentially expressed genes (DEGs) are indicated with a black box around the bar plots. (**B**) Diagrams of the *DFR* coding sequence showing color-differentiating non-synonymous SNPs in pink (> 70% frequency difference) between blue and orange *DFR-1* of *L. arvensis* and *L. monelli*, and differences between *DFR-1* (blue and orange) and *DFR-2* (orange) of both species. Gray areas indicate substrate specificity and active sites of *DFR* genes. The magnified region comparing *DFR-1* and *DFR-2* shows many NS SNPs (pink) in the largest substrate specificity region. (**C**) Volcano plot comparing the entire transcriptomes of *L. arvensis* and *L. monelli* highlighting ABP genes with significant differential expression

Transcriptome results for *Lm* petals identified 37,552 distinct genes. Of those, 336 were differentially expressed between blue and orange flowers (1.19x more genes with O > B expression; Chi-Square = 14307; p = < 2.2e-16). A total of 128 flavonoid biosynthetic pathway (FBP) genes were detected, although only five had significant differential expression, four of which had O > B expression in *Lm* (Supplementary Table S2), three of which are in the core ABP (Fig. 2C). See [35] for *La* results including the absence of any evidence of divergent homologous copies of these genes.

In comparing FBP gene expression in *Lm* to *La*, three genes (*DFR-2*, *BZ1-2* and *Caffeoyl CoA-1*) were significantly differentially expressed in both species [35] (FDR < 10^− 5^ and log_2_FC > 1; Fig. 2C, Supplementary Table S2). Two key ABP genes had high expression in orange petals and were nearly undetectable in blue petals (Fig. 2A, C). First, *DFR-2* with substrate specificity for pelargonidin [35] had high expression in orange petals (*LmDFR-2* orange mean = 157.18; *LaDFR-2* orange mean = 99.80; Fig. 2A). The second is *BZ1-2*, a gene responsible for glycosylation that stabilizes the orange pigment (*LmBZ1-2* orange mean = 2651.84; *LaBZ1-2* orange mean = 294.55; Fig. 2A). The third gene, *Caffeoyl CoA-1*, is an early gene in the ABP that functions before the first dedicated step in the ABP (not previously reported to be involved in this type of color change; Supplementary Fig. S2B). It had higher expression in blue petals of both species and was nearly undetectable in orange petals of both species - the opposite pattern as that described above for *DFR-2* and *BZ1-2*. In addition to not having known function in the biochemistry of blue and orange, this gene is also unlikely responsible for the color change because this copy of *Caffeoyl CoA* has the lowest expression of the three copies expressed in petals (Supplementary Fig. S2B).

**Fig. 3:**
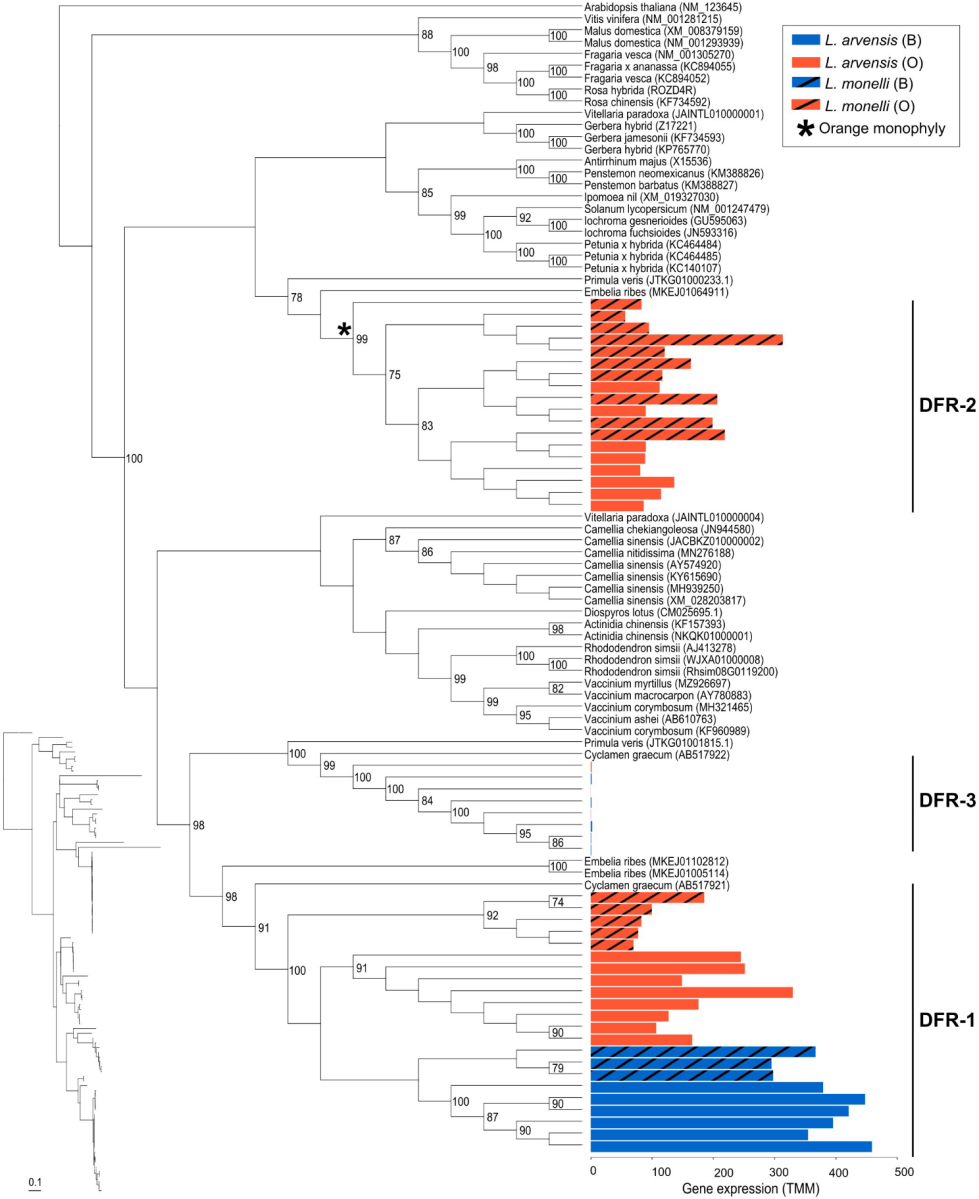
Maximum likelihood cladogram of the coding sequences of three *DFR* paralogues expressed in *Lysimachia* species. Outgroups were selected from top BLASTn hits and, when available, from the genomes of closely related species (*Camelia sinensis*, *Embelia ribes* and *Primula veris*). Outgroups are indicated by scientific name followed by Genbank Accession numbers. Bootstrap values are provided to the right of the nodes when greater than 70%. The bar plot shows the expression level (TMM) for blue and orange flowers of *L. arvensis* (solid) and *L. monelli* (black stripes). The monophyletic clade of orange petal samples for *DFR-2* is indicated with an asterisk (*). A phylogram (inset) is provided with the samples in the same order as the cladogram to compare relative branch lengths. Scale bar is in substitutions per site

Some additional ABP genes likely involved in this color shift showed significant differential expression between colors of *La* and consistent, yet non-significant trends, in *Lm*. For example, *DFR-1* and *F3’5’H* had higher expression in blue petals than in orange petals in both species, but was only significant in *La* (2.10x and 2.45x more in *La*B and 1.80x and 4.71x more in *Lm*B, respectively; Fig. 2A). Another example of significant DEG with a similar trend in *La*, but only significant with a Mann-Whitney U test in *Lm*, is *MYB4-2* which has higher expression in orange petals compared to blue petals (25.75x more in *La*O and 3.83x more in *Lm*O; Fig. 2A). Alternatively, *CHS* showed significant differential expression between colors of *Lm*, but not significant nor consistent in *La* (1.95x more in *La*B, but 2.36x more in *Lm*O; Fig. 2A) and is unlikely involved in the transition from blue to orange. Finally, a third *DFR* (*DFR-3*; Fig. 3) was detected in both species, but exhibited very low expression (maximum TMM = 1.07) indicating it is not the primary gene copy at this step in the ABP in petals (*DFR-1* and *DFR-2* have > 100x higher expression).

#### Phylogenetic analysis of ABP sequences

**Fig. 4:**
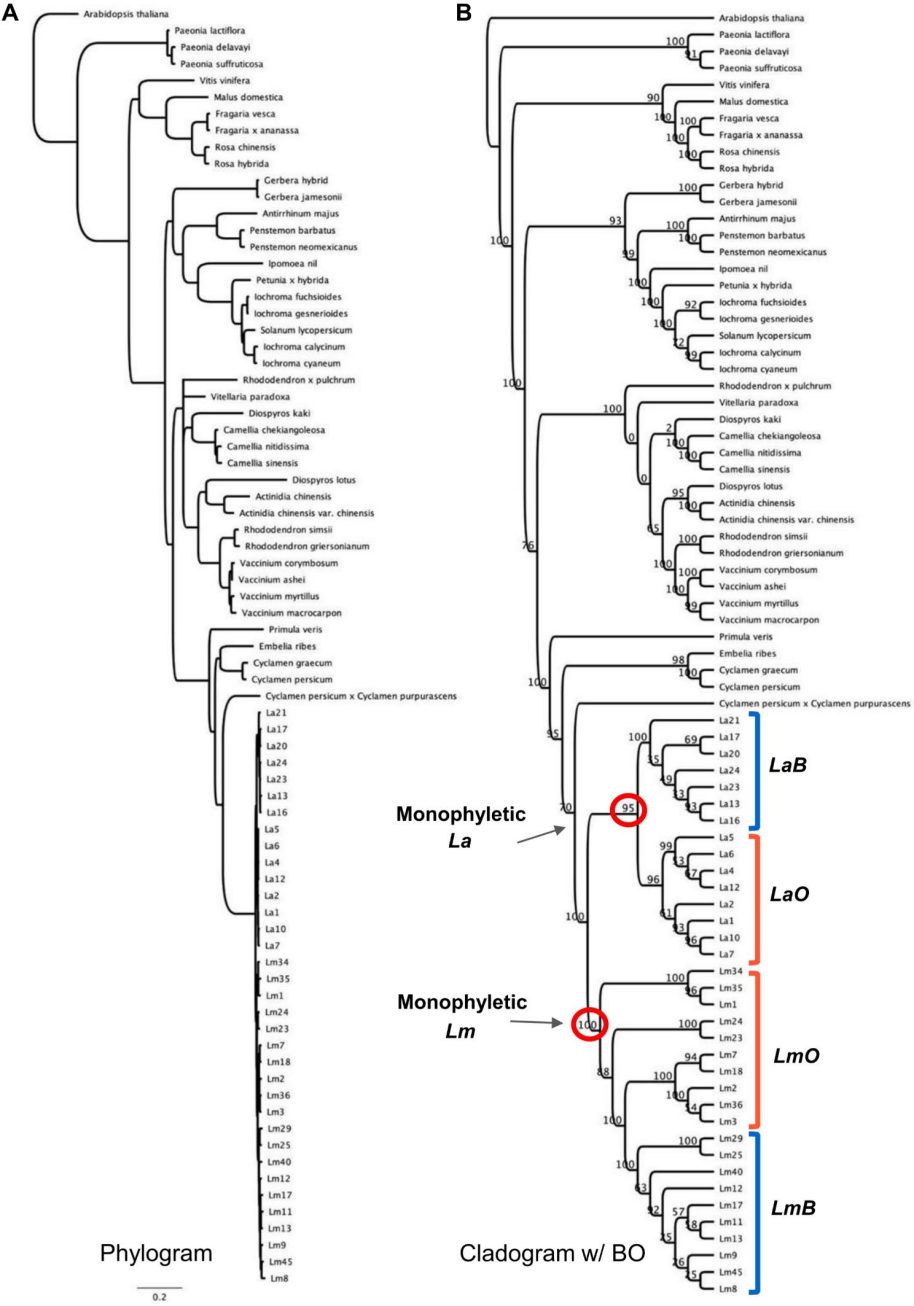
Combined maximum likelihood phylogenetic analysis of 22 ABP structural and regulatory loci. (**A**) Phylogram on the left with branchlengths proportional to the number of substitutions per site (see scale bar) and cladogram on the right with bootstrap values indicated at the nodes. (**B**) Cladogram annotated with petal color (blue (B) and orange (O) brackets). *Lysimachia arvensis* (*La*) and *L. monelli* (*Lm*) are strongly supported as monophyletic as are the color types within each species except for the paraphyly of *Lm* orange

We compared coding sequences of a total of 22 ABP genes that were either differentially expressed in one of the species or potential candidate genes responsible for flower color shift. Individual phylogenetic analyses for all 22 ABP genes (10 structural and 12 regulatory) consistently recovered a monophyletic clade of *Lysimachia* samples suggesting these were broadly orthologous comparisons (> 70% bootstrap support; Supplementary Table S3, Supplementary Fig. S2). For two key ABP genes in the transition from blue to orange (*DFR-2* and *BZ1-2*), all orange samples of both species are strongly supported as monophyletic (99% and 100% bootstrap, respectively; Fig. 3, Supplementary Table S3, Supplementary Fig. S2A). Furthermore, the regulatory locus, *bHLH12* is reciprocally monophyletic for species and within species color morphs (Supplementary Fig. S2J). In a combined phylogenetic analysis of all ABP loci, we recovered monophyletic species and monophyletic colors within each species except for paraphyletic *Lm*O (Fig. 4).

#### SNP analysis

To determine if there is an association between genotype and phenotype, we examined SNPs across the FBP loci for all samples. Genes responsible for color differences in one or both species could have non-synonymous (NS) SNPs in functional regions that correlate with color within species if causal changes are in coding regions. We detected the exact same 13 NS SNPs in *DFR-2* of orange *Lm* that differentiate it from *DFR-1* which overlap with known functional regions providing substrate specificity for pelargonidin (Fig. 2B) as we detected previously in *La* [35]. Only two loci have completely fixed differences between color morphs in both species (one NS SNP in *DFR-1* and two NS SNPs in *MYB4-2*; Supplementary Table S4), but none are located in known functional sites. Moreover, we find 16 NS SNPs that differentiate the two species in eight genes that we don’t predict are causing the color change (Supplementary Table S4). Noticeably absent from this list are the loci we have evidence are involved in the color shift (*DFR-1*, *DFR-2*, *BZ1-2*, *MYB 4 − 2*), and only one NS SNP in the extreme 5’ region of *F3’5’H* (Supplementary Table S4). Only *F3’H* showed color-specific NS SNPs (five in *Lm* vs. one in *La*), however there are even more NS SNPs between the species (seven) (Supplementary Table S4).

### Structural and regulatory gene interaction

To help discern if the shared expression differences in orange petals of the two species for *DFR-2* and *BZ1-2* are controlled by the same or different regulatory genes, we looked for patterns of correlated expression with the MYB-bHLH-WD40 regulatory complex. The rationale for investigating expression correlations is that if color has evolved via independent changes in expression (i.e. developmental convergence), then we expect gene expression correlations to be species specific. Using a hypothesis testing approach (alpha = 0.05) in both species, *DFR-2* and *BZ1-2* were significantly positively correlated with one another (*R* = 0.73, *p* = 8.8e-05 in *La*; *R* = 0.62 and *p* = 2.9e-04 in *Lm*) and with *TTG1*, a WD40 known to activate ABP structural genes (across both genes and species *p* < 0.0051; Supplementary Fig. S3). Furthermore, *DFR-1* expression is negatively correlated with *MYB4-2* expression, a known repressor of ABP structural genes (*R* = -0.68 and *p* = 2.5e-04 in *La*; *R* = -0.62 and *p* = 1.4e-04 in *Lm*; Supplementary Fig. S3). *MYB4-2* is positively correlated with *DFR-2* (*R* = 0.39 and *p* = 0.37 in *La*; *R* = 0.47 and *p* = 0.0062 in *Lm*), but non-significant after Bonferroni correction. However, we were unable to locate the EAR motif required for suppressive function [37] in *LaMYB4-2* and *LmMYB4-2* after aligning to *MYB4-1* where it was very distinctive, yet there is no differential expression was detected in *MYB4-1* (Supplementary Fig. S4).

### Reproductive isolation between *La *and *Lm*

None of the crosses performed between *La* and *Lm* produced fruits irrespective of crossing direction and flower color (*n* = 53 pollinations, see Supplementary Table S5). In contrast, morphs within species were interfertile. Between morph crosses in *Lm* produced a mean fruit-set of 47.86% ± 0.39% (*n* = 575) and a mean number of seeds per fruit of 16.25 ± 9.16 (*n* = 287) [28]. Whereas in *La*, 100% of between morph crosses produced fruits containing 16.95 ± 7.19 seeds per fruit (*n* = 97) [31].

### Climate niche modeling

The four morphs were statistically different from one another across most of the 19 BIOCLIM variables examined (Supplementary Table S6). The orange and blue *La* morphs differed significantly from each other for 17 of the 19 climate variables, and the two *Lm* morphs differed for nine variables. The two orange morphs differed from one another for 16 variables and the two blue morphs differed across 11 variables.

Nine variables were significantly different between the two blue morphs and between the two orange morphs of both species. Of these, two temperature variables differed in parallel (e.g., for “isothermality” orange morphs showed lower values than blue morphs for both species) (Supplementary Fig. S5A), while three temperature and four precipitation variables differed in opposing directions (e.g., for “annual precipitation” orange *La* were wetter than blue *La*, but orange *Lm* were drier than blue *Lm*) (Supplementary Fig. S5C).

The logistic regression results showed that the two best models using AIC did not employ any of the same variables. The best model for *La* included (in decreasing order of importance based on standardized coefficients) precipitation seasonality, isothermality and temperature seasonality, while the model for *Lm* consisted of precipitation of the wettest month and mean temperature of the wettest quarter (Supplementary Table S7).

Since the ancestral state in *Lysimachia* is blue flowers, we hypothesized that blue would be less likely to diverge in their climate niche between the two species compared to orange morphs of the two species. We tested this with a Monte Carlo procedure that randomized colors within species and then compared observed climatic differences with differences from the null distributions. Across the 19 BIOCLIM variables, blues were more divergent between the two species than expected by chance for only one variable while orange samples were more divergent between the two species for 15 variables (~ 79% of variables studied), and neither color morph was significantly different for three variables (Supplementary Table S8).

## Discussion

The color morphs of these two *Lysimachia* species have nearly identical biochemical and molecular underpinnings as measured thus far. The evolution of orange pelargonidin 3-glucoside from blue malvidin 3-rhamnoside requires (1) redirecting ABP flux down the pelargonidin branch and (2) stabilizing the newly formed orange anthocyanidin with glucose instead of rhamnose. Both steps are accomplished in the same way in both species - via the recruitment of orange-specific paralogues that are undetectable in transcriptomes of blue petals. In fact, the copy of *DFR* unique to orange petals (*DFR-2*) has nearly the same overall gene expression levels as the shared copy (*DFR-1*), but its expression is undetected in blue petals of both species. Furthermore, *DFR-2* of both species contain the same 13 NS SNPs in the substrate specificity region that differentiate it from *DFR-1*, which likely allows it to outcompete *F3’5’H* (and *F3’H*) for dihydrokaempferol thereby shunting flux down the pelargonidin branch leading to orange petals [38–40]. The substantial genetic distance between *DFR-1* and *DFR-2* suggests that this duplication predated the genus and maybe even the Primulaceae family – a molecular toolkit deployed when pelargonidin provides a selective advantage over malvidin.

The coordinated changes in ABP gene expression are like other blue to red flower color shifts that involve the recruitment of substrate specific *DFR* copies with correlated downregulation of the alternative side-branch gene expression (e.g., *F3’H* and *F3’5’H*) [41, 42]. We also find decreased expression in *F3’5’H* (but not *F3’H*) in orange petals of both species, however, this is only significant in *La* due to high expression variation among the blue *Lm* samples (Fig. 2). Regardless, the shared expression and nearly identical coding sequences of orange *DFR-2* in *La* and *Lm* are strong evidence of a single origin that predates this speciation event and has persisted to the present in these distinct lineages.

Similarly, orange petals employ an orange-specific glycosyltransferase (*BZ1-2*), undetected in blue petals, glycosylating the orange pigment in the same way in both species. The shift from blue to orange is correlated with a dramatic increase in expression of the glycosyltransferase *BZ1-2* (similar to the *DFR*s mentioned above), which is likely involved in the stability, solubility, storage and biological activity of this particular anthocyanin [43]. There are no NS SNPs when comparing *BZ1-2* between orange samples of *La* and *Lm* (same evolutionary history), however two *BZ1* paralogues are highly divergent (> 60% nucleotide divergence) and unalignable indicating a relatively ancient duplication event. The orange-specific *BZ1-2* of both species contains NS SNPs correlating with glucose-specificity as characterized in *Vitis vinifera* and *Medicago truncata* [44, 45]. In particular, there are 20 NS SNPs distinguishing these two paralogues in the PSPG-box, a conserved 44 amino acid region found in all plant UFGTs [46] that likely confers glucose-specificity of *BZ1-2*. We infer that the two paralogues likely have different functions regarding which sugar they add and their efficiency in doing so [47], but within a color morph for both species, their sequence similarity suggest they perform the same function. In contrast to *BZ1-2*, *BZ1-1* has variable expression in both color morphs - *La* has significantly higher expression in blue than orange as expected, but mean expression is similar in blue and orange *Lm* (but not significant). Regardless of whether there is compensatory downregulation in orange petals of *BZ1-1*, we know that *BZ1-2* has 10-50x higher expression in orange and we predict that it has higher efficiency in adding glucose to pelargonidin of orange petals than *BZ1-1* [47]. Although there are other examples of closely related species exhibiting the same flower color because they accumulated similar major categories of anthocyanidins (i.e. aglycones) [48–50], the specific anthocyanins they accumulate are generally distinct due to the enormous diversity of biochemical decorations in the flavonoids [51, 52]. In these two *Lysimachia* species, orange morphs use the same *3GT* correlated with the predominant glycosylation of pelargonidin suggesting a unique transition event from blue to orange state.

If *DFR-2* and *BZ1-2* are similarly upregulated in orange *La* and *Lm*, we predict they will be controlled by the same regulatory gene(s) in both species. In fact, expression of *DFR-2* and *BZ1-2* in orange petals of both species is positively correlated with the same regulatory gene *TTG1* (Supplementary Fig. S3), which has been found to form MBW complexes with MYBs and bHLH genes to regulate the expression of the late ABP genes [53]. Looking at the sequence of this regulatory gene, there is only one color differentiating NS SNP in *La* and none in *Lm* strongly suggesting another trans-acting regulatory gene is likely responsible for the differential expression of *TTG1* in blue and orange morphs of these two *Lysimachia* species. However, *MYB4-2* has two NS SNPs shared by both species that positively correlates with *DFR-2* expression (Supplementary Fig. S3).

For both *Lysimachia* species, *DFR-1* expression in orange petals is negatively correlated with *MYB4-2* expression (Supplementary Fig. S3). Moreover, in *La* this increase of *MYB4-2* is correlated with a decrease in *F3’5’H* in orange petals, facilitating the redirection of ABP flux to the pelargonidin branch of the pathway. In *Arabidopsis thaliana* and *Vitis vinifera*, *MYB4* represses anthocyanin biosynthesis either through direct binding to the promoter regions of ABP genes (*ANS*, *DFR* and *UFGT*) or by displacing the MYB activator in the MBW complex [54, 55]. However, we were unable to locate the EAR motif required for suppressive function [37] in *LaMYB4-2* and *LmMYB4-2*, which is easily identifiable in its non-differentially expressed paralogue *MYB4-1* (Supplementary Fig. S4), suggesting that *MYB4-2* may not have the same capability to repress expression of *DFR-1* and *F3’5’H* in orange petals as described in other systems [37]. Therefore, although we found a positive correlation of *TTG1* expression with *DFR-2* and *BZ1-2*, and a negative correlation of *MYB4-2* with *DFR-1* and *F3’5’H*, final conclusions regarding the roles of these regulatory genes in these flower color polymorphisms will require further experimental molecular genetic dissection.

Previous experimental work and our climate niche modelling indicate that there may be pleiotropic effects of petal color. Orange morphs of both species are found in locations with colder winters than blue morphs (Supplementary Fig. S5A), even though for *La* both morphs can be found in sympatry in the central portion of the species range whereas in *Lm* the two color morphs are completely allopatric (Fig. 1A). However, the two morphs appear to respond in opposing directions to primarily moisture-related climate niche variables (Supplementary Fig. S5C). Orange *Lm* is found in habitats with a wide range of precipitation while orange *La* is found in wet habitats. Although the climate niche of the blue morphs of the two species are often distinct, the orange samples are clearly driving these orthogonal responses to precipitation. Why a biochemically and genetically similar polymorphism shared between different species would have contrasting ecological side-effects remains unclear. Previous experimental work in *La* shows that blue-flowered individuals (containing rhamnose stabilized malvidin) inhabit environments with lower precipitation and higher solar radiation than orange-flowered plants with glucose-bound pelargonidin, potentially linking abiotic stress with differential glycosylation [28, 29]. If *BZ1-2* is only found in orange petals and is glucose-specific, then does the type of sugar confer physiological or ecologically-relevant adaptations in *Lysimachia*? The coupling of rhamnose to malvidin found in *Lysimachia* also explains the blue color of *Petunia hybrida* [56], *Lobelia erinus* [57], and *Parochetus communis* [58], and is known to provide stress tolerance (e.g. UV response) when rhamnose is bound to flavonoids [59, 60]. However, in *Lm*, the opposite is true for several climate niche parameters, especially with regard to precipitation (Supplementary Table S7, Supplementary Fig. S5C). This flower color polymorphism example appears to be driven by non-pollinator agents of selection – specifically the distinct climatic niches in each species, especially those involving precipitation variables [28–30], yet does not appear to be maintained by negative frequency dependent selection, the most common mechanism maintaining polymorphisms over long periods of time in other species of plants and animals.

**Fig. 5:**
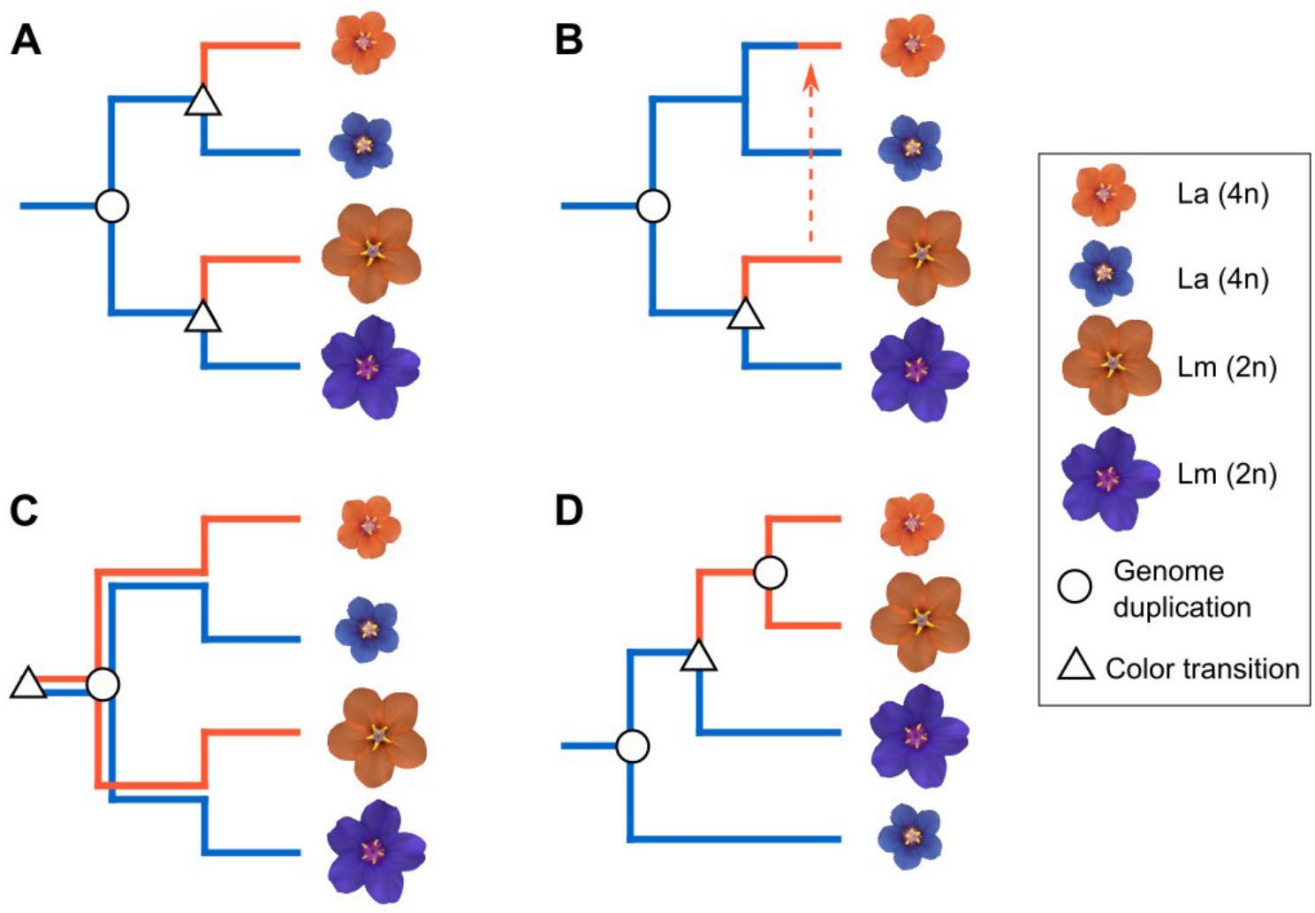
Four evolutionary hypotheses of petal color evolution in *L. arvensis* and *L. monelli*. Assuming the ancestral state is blue and diploid, the most parsimonious color shifts are indicated by a triangle and ploidy changes are indicated with a circle (placed at the node since they likely drive divergence). In (**A**), convergent evolution of orange petals is coupled with a single origin of the tetraploid lineage predicts independent and distinct molecular causes for the color change in each lineage. In (**B**), a single origin of orange petals in *L. monelli* followed by introgression to *L. arvensis* requires only one shift to orange and one polyploid event but requires the lineages to be able to hybridize. In (**C**), a single origin of orange in the common ancestor creates an ancestral polymorphism that transcends the speciation event and persists to the present in both species, requiring one polyploidy event. In (**D**), non-monophyletic species allow for a single origin of orange petals but requires independent origins of the tetraploid lineages. See Material and Methods for more details

Given all these comparisons, there are many similarities (and some differences) between color morphs of *L. arvensis* and *L. monelli* that can be used to evaluate the alternative hypotheses describing their origin (Fig. 5; Supplementary Table S9). The same biochemical compounds and molecular causes (expression, SNP variation, and correlation of structural and regulatory key ABP genes) found in the orange morph of both species suggest that the orange color likely evolved only once. Although the exact mutation(s) responsible for the color shift in each species awaits further genetic dissection, this first multiscale approach provides very little support the convergent evolution hypothesis (Fig. 5A; Supplementary Table S9) since we would expect distinct molecular causes [48, 49, 61]. Moreover, the chances of a single shift from blue to orange followed by introgression into the other species (Fig. 5B) as clearly documented in the *Diplacus* (*Mimulus*) *aurantiacus* complex [62, 63] is unlikely for *Lysimachia* species for three reasons. First, although the two species co-occur, hybrids with intermediate flower size and plant size have never been observed in the present [64] unlike in the *Diplacus aurantiacus* complex where hybrid zones are well documented. Second, numerous attempts to cross the two species reveal complete reproductive isolation (Supplementary Table S5), presumable because of the ploidy differences – whereas the members of the *Diplacus aurantiacus* complex are at least partially interfertile [62]. Third, the 22 ABP gene phylogeny (Fig. 4) showed monophyly of species, and with an orange introgression event we would expect ABP genes of orange morphs of both species to be more closely related to one another when compared to different morphs of the same species. In the same way, the species monophyly (Fig. 4) and the lack of fruit production between species, but interfertility between morphs within species, also refutes the non-monophyly of species hypothesis [32] (Fig. 5D). In contrast, the evidence shown in this work supports the ancestral polymorphism hypothesis (Fig. 5C). Given that case, both blue and orange individuals would have persisted through the speciation process including polyploidy, loss of self-incompatibility, significant reduction in flower size, and a shift from perennial to annual, which is the most parsimonious explanation for *La* and *Lm* species given the current evidence. The persistence of polymorphisms across species has been documented in a few exceptional animals [14] and in even fewer cases as the trans-specific shared polymorphism for self-incompatibility alleles [2], and potentially anther-color [7]. In that sense, this would be the first example in plants with regards to a petal color polymorphism persisting across species that diverged 2.9–8.1 Mya (mean = 5.2 Mya) [32].

Since flower color can confer reproductive isolation [65, 66], these intraspecific color polymorphisms may represent the initial stages of speciation. Under this scenario, *La* represents an incipient stage in the process (100% interfertility between the two color morphs) whereas *Lm* may be further along in the speciation continuum (< 50% inter fertility between the two color morphs). However, the mechanism by which these color differences confer reproductive isolation is unclear since there is no detectable pollinator preference [27]. These reproductive isolation comparisons are consistent with the geography of speciation for these two lineages - *La* has sympatric populations of orange and blue color morphs whereas *Lm* color morphs are always allopatric – providing additional evidence that *Lm* may be further along in the process. We suggest the corolla color variation confers a non-pollinator adaptation to one or more climate variables [29] which may eventually drive complete reproductive isolation between the morphs of each lineage.

However, there is still some lingering evidence for alternative hypotheses that does not allow us to make an unequivocal conclusion (Supplementary Table S9). Further analysis of gene function, enzyme kinetics and genetic association must be conducted before any final conclusions can be made regarding the molecular causes and ecological consequences of this flower color variation. However, the data presented herein lean toward a single biochemical and molecular footprint shared by both species. Additional experimental approaches (genetic dissection, transformation, knock-out, *DFR* specificity enzyme assays, etc.) promise to identify the type, number, and order of mutations responsible for these flower color changes, yet given the phenotypic, biochemical and transcriptome similarities described here, we have yet to find any strong evidence rejecting the ancestral polymorphism hypothesis.

## Methods

### Population sampling

We collected *Lm* plants from 10 blue-flowered populations from southern Spain and Portugal, and from 10 orange-flowered populations from northern Spain, Morocco and Italy. Mercedes Sánchez-Cabrera, Francisco J. Jiménez-López, Montserrat Arista, and Pedro L. Ortiz identified the plant material used in this study. Voucher specimens were deposited in the University of Seville Herbarium (SEV, Supplementary Table S10). See *La* sampling information in [35].

For RNA-Seq analysis we sampled all five petals per flower from a mean of 19 flowers per plant (10–24 flowers) from ten blue- and ten orange-flowered plants of *Lm*, from seven and five populations respectively. The bullseye at the petal base was avoided. All samples were taken from first-day anthesis flowers and immediately, petals were flash frozen and stored at -80ºC. For flavonoid profiling of *Lm* petals (see Supplementary Material and Methods S1), we collected 10 blue and nine orange samples (two to five flowers without the bullseye per sample; Supplementary Table S10). RNA-seq data and flavonoid profiling of *La* petals were obtained from [35].

### UV-Vis petal spectra

Reflectance spectra of the adaxial surface of 75 blue and 50 orange petals of *Lm* were captured using a JAZ A1465 double-beam spectrophotometer (Ocean Optics, Florida, USA). The deuterium-tungsten light source provided reflectance between 300 and 700 nm. Reflectance spectra data of *La* was obtained from [27].

We tested the UV-vis spectra for the relative amounts of variation in two tests (1) between color morphs within a species versus (2) between species within a color morph using permutational multivariate analysis (PERMANOVA test) [67]. For each test, we computed the Euclidean distances based on the difference in reflectance at each wavelength between all pairs of individual spectra and conducted permutational MANOVA tests [68] to assess statistical differences (9999 permutations) making quantitative comparative statements between the R^2^ values of the two tests (adonis2 in “vegan” package [69] in R v4.0.0 [70]). We visualized the distances between the spectra using metaMDS in “vegan” in R [70] with 500 tries.

### RNA extraction and reads filtering

Collected petals (see Population sampling section) were homogenized using a mortar and pestle. Total RNA was then extracted following the Qiagen RNeasy Plant Mini Kit protocol (Qiagen, Germany) with the addition of PEG 20,000 mol. wt. (550 µl, 2%) [71] before the first filtering step. The addition of PEG was essential to achieve reasonable RNA concentrations for library preparation and sequencing. RNA samples were stored at -80ºC until further analysis. RNA concentration and purity was initially assessed with a Nanodrop Nd-1000 (ThermoFisher) and agarose gel, and then confirmed with a Bioanalyzer (Agilent, Santa Clara, CA, USA) before sequencing. Individual libraries were barcoded, multiplexed and sequenced as 150 bp paired-end reads using two lanes on an Illumina Hi-Seq 2000 (Illumina, San Diego, CA, USA) through Novogene (Beijing, China). Raw paired-end Illumina reads were assessed for quality using FastQC [72] and were processed using Rcorrector v1.0.4 [73] to correct random sequencing errors. Then, reads were trimmed with Trimmomatic v0.39 [74] to remove any read containing bases with Phred scores lower than 20, low quality reads less than 50 bp long, and any adapter or other Illumina-specific sequences that were still present (Supplementary Table S11).

### Identification of differentially expressed genes (DEGs)

To determine DEGs for *Lm*, we used the *La* transcriptome assembly with known ABP genes identified [35] as reference for mapping *Lm* RNA-Seq samples with Bowtie2 software [75]. We calculated Trimmed mean of M-values (TMM, mean of log-expression ratios) [76] for each gene using RSEM software [77]. Then, with “edgeR” package [78] in R we determined statistically significant differentially expressed genes (DEGs) between blue and orange petal samples, applying the conservative thresholds for DEG identification: the false discovery rate (FDR) less than 10^− 5^, and the expression difference threshold greater than one log_2_ fold-change (log_2_FC) [35]. To keep biologically interesting genes for differential expression analysis, we considered those genes with more than one count per million (CPM) in a minimum of four samples. Some regulatory genes have very low expressions and magnitudes were unreliable. For those loci, we performed the non-parametric Mann-Whitney U test to test for differential expression.

### Genes and isotigs selection for analysis and correlated expression analysis

We selected 22 ABP color differentiating structural and regulatory genes with differential expression between colors in either both species, just in one species or not differentially expressed but was a copy of a DEG (see Results section). We established a criterion to select one isotig per gene: (1) samples with less than 20% ambiguous nucleotides in the CDS, (2) samples with more than 100 reads mapped to the sequence, (3) samples with the longest CDS, (4) samples with higher expression. Finally, we selected the isotig which was present in a higher number of samples after applying the criteria. We tested for correlated expression among the 22 structural and regulatory genes in both species using Kendall correlations and applying a Bonferroni corrected p-value of 0.001 to reduce the likelihood of falsely reporting a correlation when none exists. These analyses were performed in R.

### Phylogenetic analyses and non-synonymous SNP identification

We conducted phylogenetic analyses on the selected isotigs for all ABP structural and regulatory genes. We followed the methods described in [35] to map to reference the reads using Geneious v9.0.4 [79]. For the outgroups, we used top BLASTn hits (filtering for *Cyclamen* sp., *Camellia* sp., *Vaccinium* sp., *Actinidia* sp. and *Rhododendron* sp. when available) and, when possible, top tBLASTx from *Camellia sinensis*, *Embelia ribes* and *Primula veris* genomes. We reconstructed the relationships using the RAxML 7.2.8 plug-in from within Geneious [79], searching for the maximum likelihood tree with 1000 bootstrap replicates. We added normalized expression values (TMM) to the phylogenetic trees with “ggtree” [80] and “phytools” [81] packages in R. We concatenated alignments of all ABP structural and regulatory loci and performed a combined phylogenetic analysis using the same methodology as above but partitioning the data by locus and determining the best fit model of nucleotide substitution.

We calculated the NS SNP rate between blue and orange petals of each species, with a cutoff of 75% difference between colors. We used the isotigs selected for NS SNP search. We focused on genes with orange monophyly to either one or both species, because orange is derived from blue and therefore, we expect genes responsible for the blue to orange shift to show a single common ancestor for the orange samples.

### Reproductive isolation between *La* and *Lm*

To determine reproductive isolation between *La* and *Lm*, we performed controlled crosses in plants from different populations grown in glasshouse (See population information in Supplementary Table S5). Flowers were emasculated before anthesis, and hand-pollinations were made during the first day of anthesis within and between species and color morphs. Pollinated flowers were left to set fruits and the fruit production and the number of seeds per fruit were counted.

### Climate niche modeling

To test whether the color morphs of each species differed ecologically across their ranges, we constructed an occurrence dataset using a combination of personal observations and iNaturalist records (inaturalist.org; manually verified for species identification and color; Supplementary Fig. S6). The dataset comprised 4287 records. All 19 BIOCLIM variables were then extracted for each record based on GPS coordinates at the highest available spatial resolution (30 s) [82]. Occurrences with no climate data and duplicated occurrences (multiple occurrences of the same species with the same flower color within a pixel) were deleted. There were 3309 occurrences in the final dataset (*La* blue = 641, *La* orange = 1941, *Lm* blue = 662, *Lm* orange = 65). We tested for univariate climatic differences among the four morphs, whether similar climate variables were most important for separating color morphs in the two species, and whether the two orange morphs were more divergent than the two blue morphs across the 19 BIOCLIM variables (see Supplementary Material and Methods S2).

### Differentiating the hypotheses of the shared flower color polymorphism

To differentiate the four hypotheses (Fig. 5) we used the following logic. First, the convergence hypothesis predicts separate origins of the blue to orange color shift which would produce separate clades of orange samples for both species. Under this hypothesis, and with enough data, *La* and *Lm* should be reciprocally-monophyletic. The introgression hypothesis involves a single origin of the color change in both species (after the speciation event) which would appear as very close relationships for the color differentiating locus between orange *Lm* and *La* since they are the same gene, just shared across species boundaries (for the color causing gene(s) the species should not be monophyletic), yet for non-color causing loci, species may be monophyletic. However, the species must be interfertile. Third, the ancestral polymorphism hypothesis produces a single origin of the color change (before the speciation event) creating separate monophyletic blue and orange clades. Finally, the non-monophyletic species hypothesis [32], which would represent a monophyletic orange clade (across species boundaries) – a topology expected for all loci, not just those contributing to the color shift. We have summarized our results in light of these four alternatives in Supplementary Table S9.

## Electronic supplementary material

Below is the link to the electronic supplementary material.

Supplementary Material 1

## Data Availability

The datasets supporting the conclusions of this article are available in the Genbank repository (BioProject: PRJNA1110038).
