# Supplementary material for: A multiscale approach to understanding the shared blue-orange flower color polymorphism in two Lysimachia species
Source: BMC Plant Biol. 2024 Sep 30;24:905. doi: 10.1186/s12870-024-05481-y (PMC11441164; doi:10.1186/s12870-024-05481-y)
Supplement: Supplementary file 1 — Supplementary Material 1 [file 12870_2024_5481_MOESM1_ESM.docx]

**Supplementary material**

**A multiscale approach to understanding the shared blue-orange flower color polymorphism in two *Lysimachia* species**

Mercedes Sánchez-Cabrera*, Eduardo Narbona, Montserrat Arista, Pedro L. Ortiz, Francisco J. Jiménez-López, Amelia Fuller, Benjamin Carter, and Justen B. Whittall

***Corresponding Author:** mercesc@us.es

**This PDF file includes two supplementary material and methods sections, six figures and 11 tables.**

**Supplementary Material and Methods S1. Identification of flavonoid compounds.**

Flavonoids were extracted by placing petal samples (see Population sampling section; Supplementary Table S9) in 1.5 mL microfuge tubes containing 500µl of MeOH with 1% HCl and stored at -80°C. Homogenization was performed with a BeadBeater using 3 x 3.2 mm steel balls for 30 seconds, then centrifuged for 10 min to pellet cellular debris. Samples of the supernatant were loaded on a Dionex UltiMate3000 ultra-high pressure liquid chromatography system equipped with a diode array detector and connected to a Thermo Fleet LCQ mass spectrometer (Thermo-Fisher, Waltham, MA USA). Data were analyzed using Xcalibur software (Thermo Fisher Scientific). Flavonoid identification was based on the compound's retention time, UV-Vis spectra and whenever possible, chromatographic comparisons with authentic standards: quercetin, luteolin, kaempferol, isorhamnetin, malvidin, pelargonidin, cyanidin and delphinidin (Extrasynthese, Genay, France), previously reported for other *Lysimachia* species (see Sánchez-Cabrera et al., 2021). Relative contents (%) of the primary anthocyanins of each color morph sample, based on the total area under the curve, were calculated and used in statistical comparisons between the two species applying a Mann-Whitney U test in R.

References:

Sánchez-Cabrera M, Jiménez-López FJ, Narbona E, Arista M, Ortiz PL, Romero-Campero FJ, et al. Changes at a critical branchpoint in the anthocyanin biosynthetic pathway underlie the blue to orange flower color transition in *Lysimachia arvensis*. Front. Plant Sci. 2021;12:247.

**Supplementary Material and Methods S2. Climate niche modeling**.

To test for univariate climatic differences among the four morphs, a separate Kruskall-Wallis test with a post-hoc Dunn test (non-parametric analogs of ANOVA with post-hoc Tukey’s test) was applied to each variable. Significance tests used Bonferroni adjustment for 76 comparisons (19 climate variables across four species/flower color combinations). To determine whether similar climate variables were most important for separating color morphs in the two species, logistic regression models were built for each species on a subset on non-correlated climate variables. To test whether the two orange morphs were more divergent than the two blue morphs across the 19 BIOCLIM variables, a randomization procedure was used.

To determine whether similar climate variables were most important for separating color morphs in the two species, logistic regression models were built for each species on a subset on non-correlated climate variables. First, strongly correlated climate variables were removed and a dataset of five variables that were both relatively uncorrelated and putatively biologically meaningful based on field observations and experimental manipulations (Arista et al. 2013; J. Jiménez-Lopez et al., unpublished data) were retained: precipitation of wettest month, precipitation seasonality, isothermality, temperature seasonality and mean temperature of wettest quarter. Data were normalized using the ‘normalize’ procedure in the ‘decostand’ function from the vegan R package (Oksanen et al. 2019) to facilitate later comparison of regression coefficients. Best fit models were selected using AIC scores derived from the ‘dredge’ function from the MuMIn R package (Bartón, 2020).

To test whether the two orange morphs were more divergent than the two blue morphs across the 19 BIOCLIM variables, a randomization procedure was used. The specific null hypothesis was no difference between the distance between the two orange morph means and the distance between the two blue morph means. To build a null model for each of the 19 variables, species identities were retained but color morphs were randomized 999 times. For each distribution, one tail indicated that differences between orange populations was greater while the other tail indicated greater differences between blue populations. A two-tailed test indicated whether observed orange differences were greater than expected, whether blue differences were greater than expected or whether neither color was more divergent.

References:

Arista M, Talavera M, Berjano R, Ortiz PL. Abiotic factors may explain the geographical distribution of flower colour morphs and the maintenance of colour polymorphism in the scarlet pimpernel. J. Ecol. 2013;101:1613–1622.

Bartón K*.* MuMIn: Multi-Model Inference. 2020

Oksanen J, Blanchet FG, Friendly M, Kindt R, Legendre P, McGlinn D, et al. vegan: community ecology package. 2019.

**Supplementary Figure S1.** NMDS plot of UV-Vis reflectance spectra for blue and orange petalled individuals of *L. arvensis* (LYAR) and *L. monelli* (LYMO). Statistical analysis was assessed with permutational manova (Anderson, 2001) with the following results (1) restricting permutations within color (comparing across species), R^2^ = 0.15113, F = 41.662, p < 1e-04 vs. (2) restricting permutations within species (comparing across colors), R^2^ = 0.74999, F = 701.98, p < 1e-04. Very similar results were found after applying a brightness correction – subtracting the minimum reflectance value of each sample from all wavelengths (not shown).

*BZ1-2 and -3*


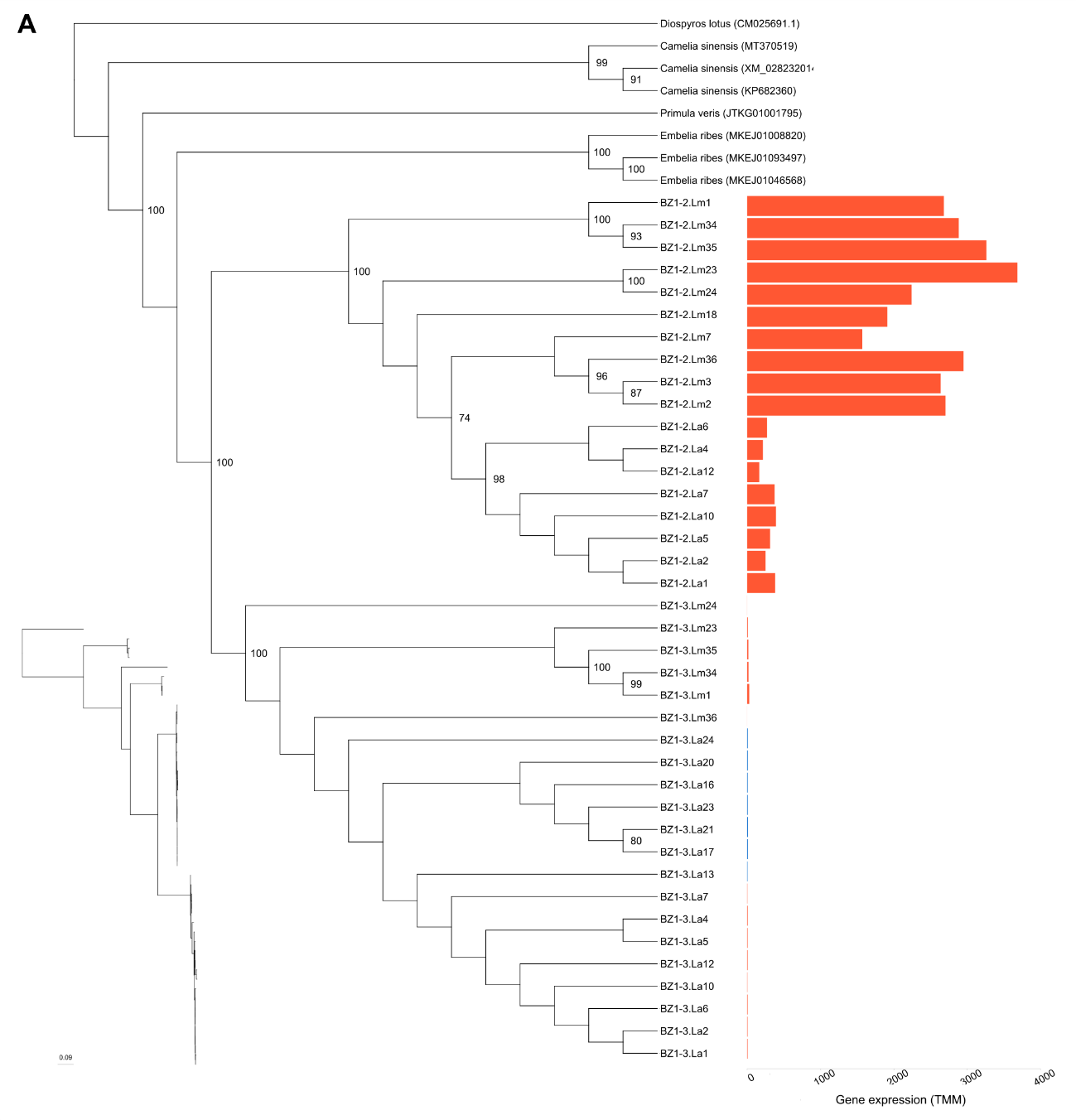


*Caffeoyl CoA-1*,*-2* and *-3*.


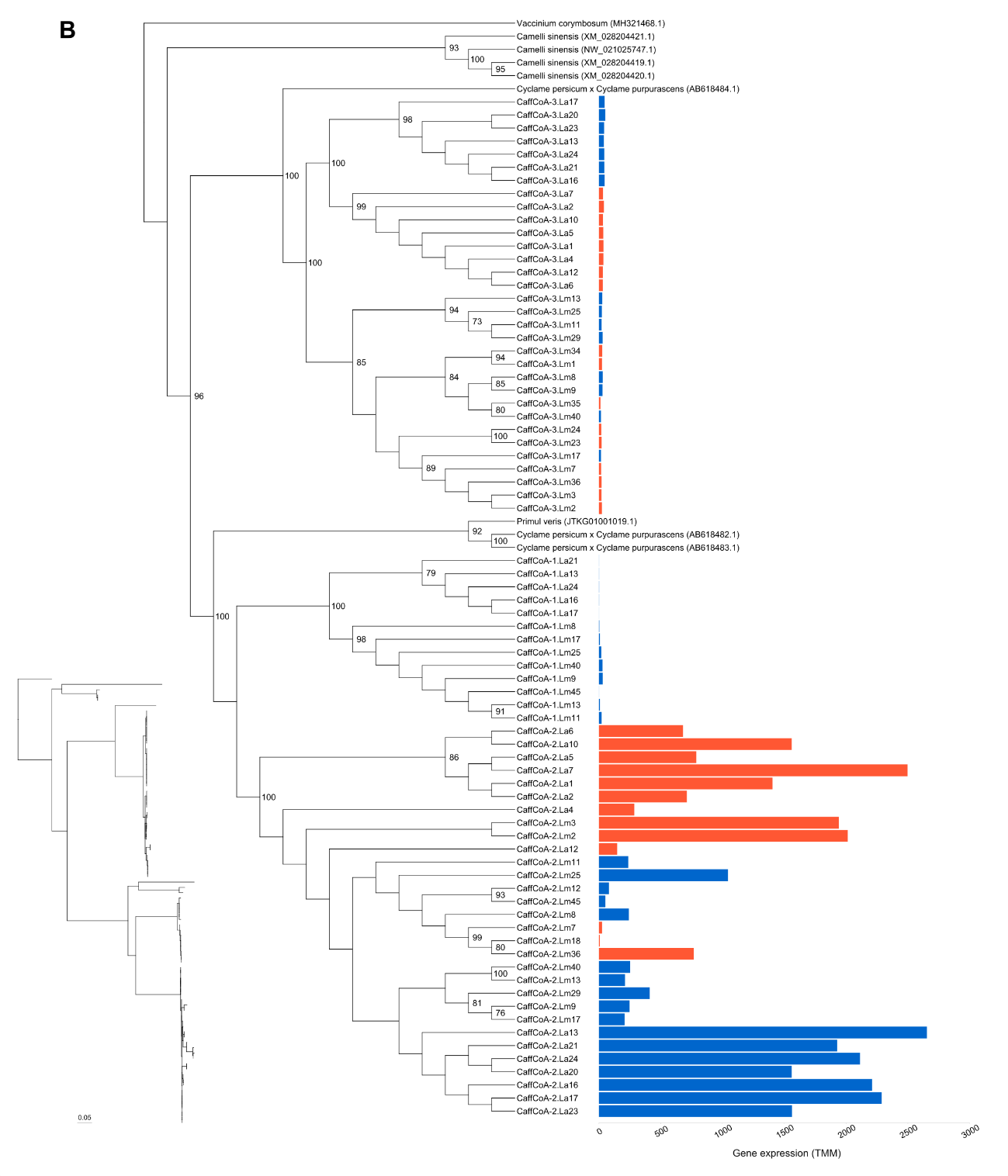


*CHS*


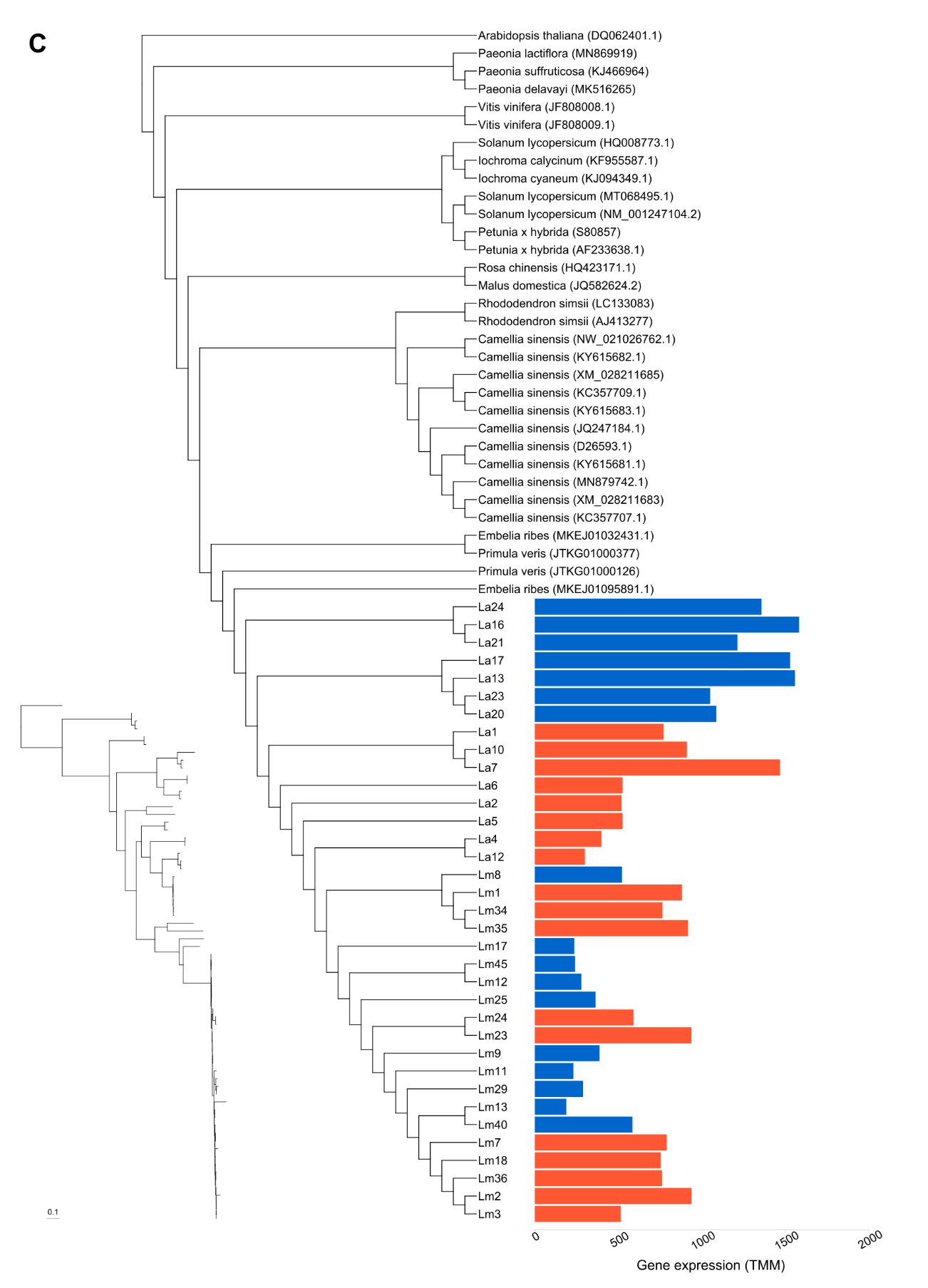


*F3’5’H*


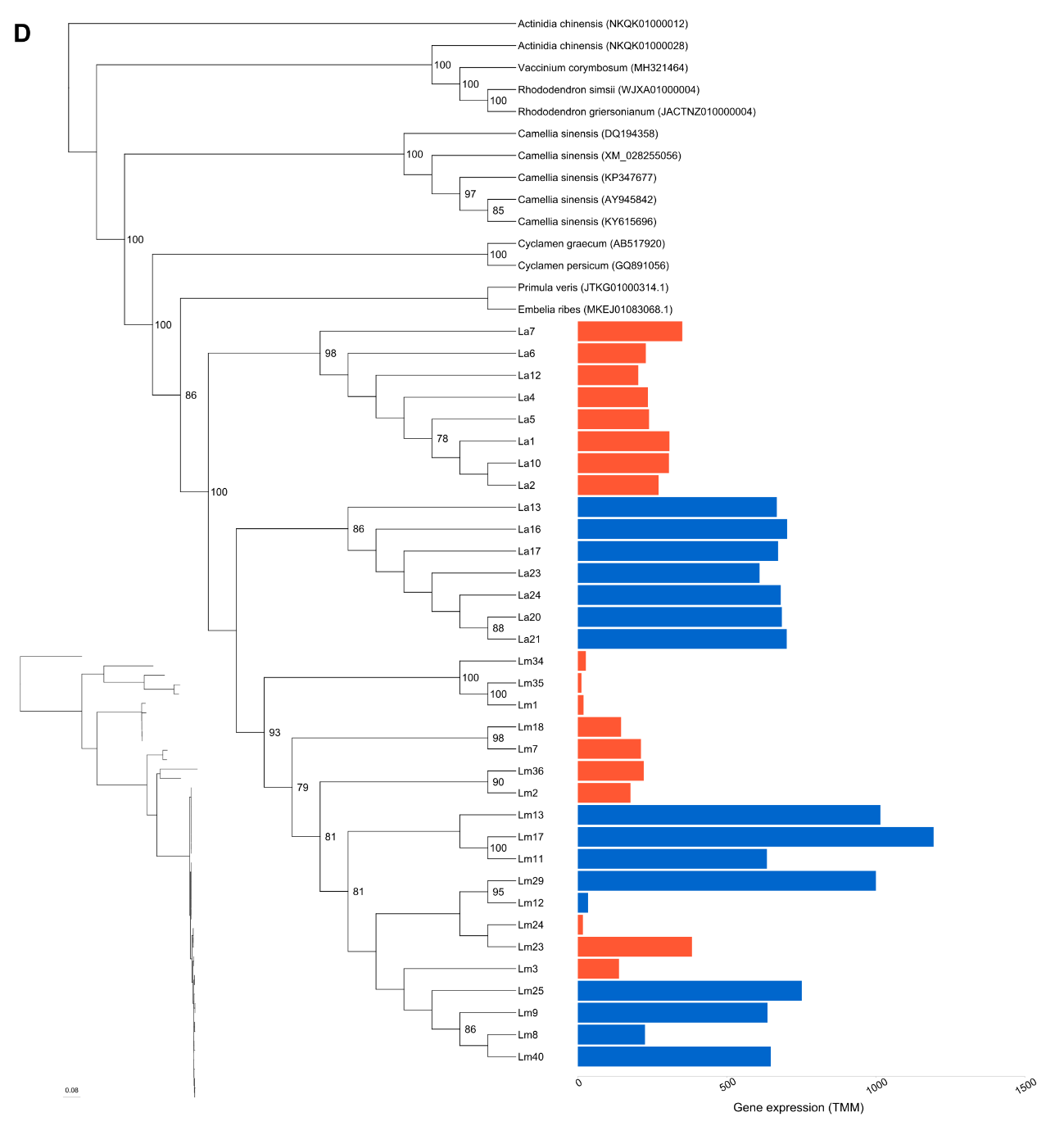


*F3’H*


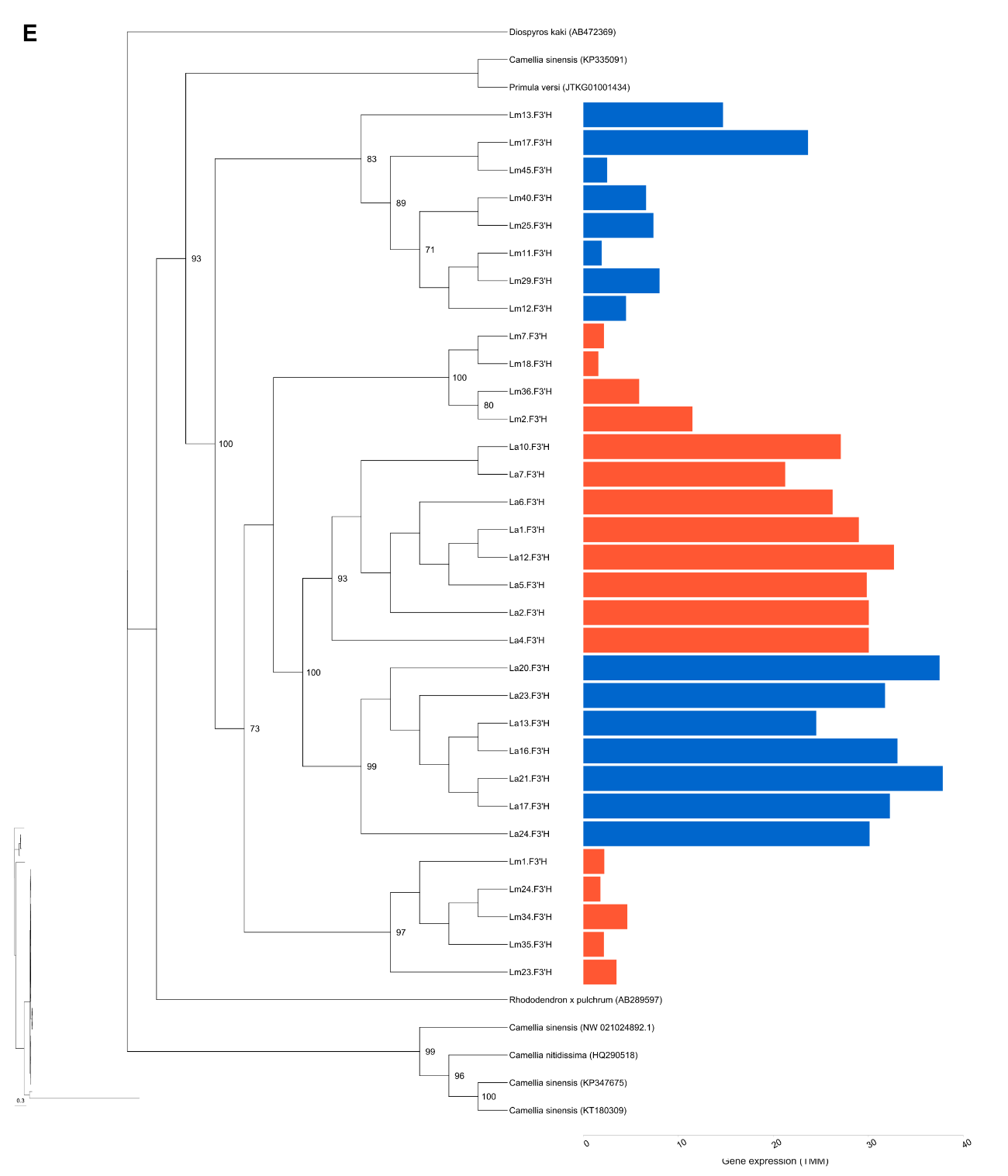


*AN11a*


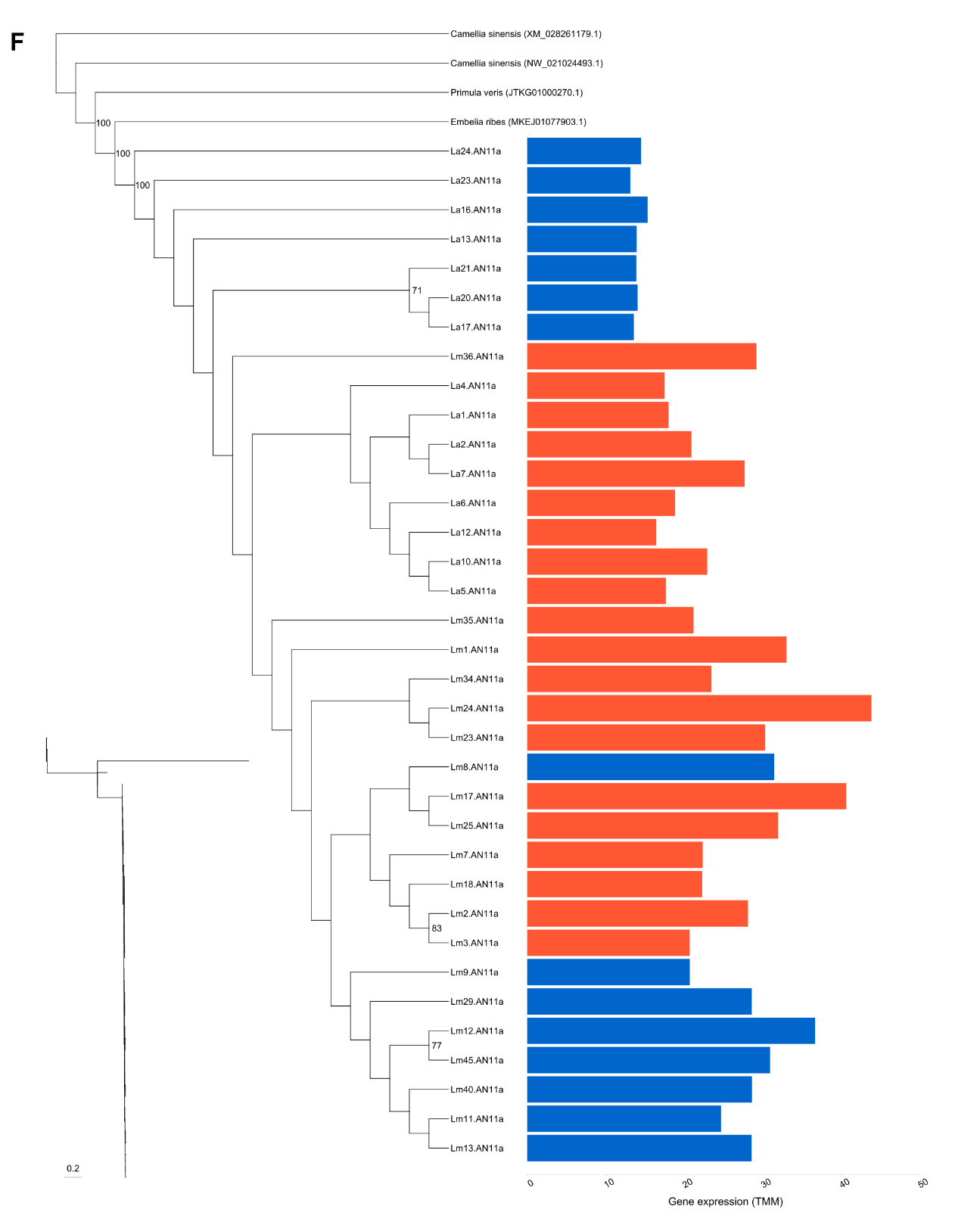


*bHLH1*


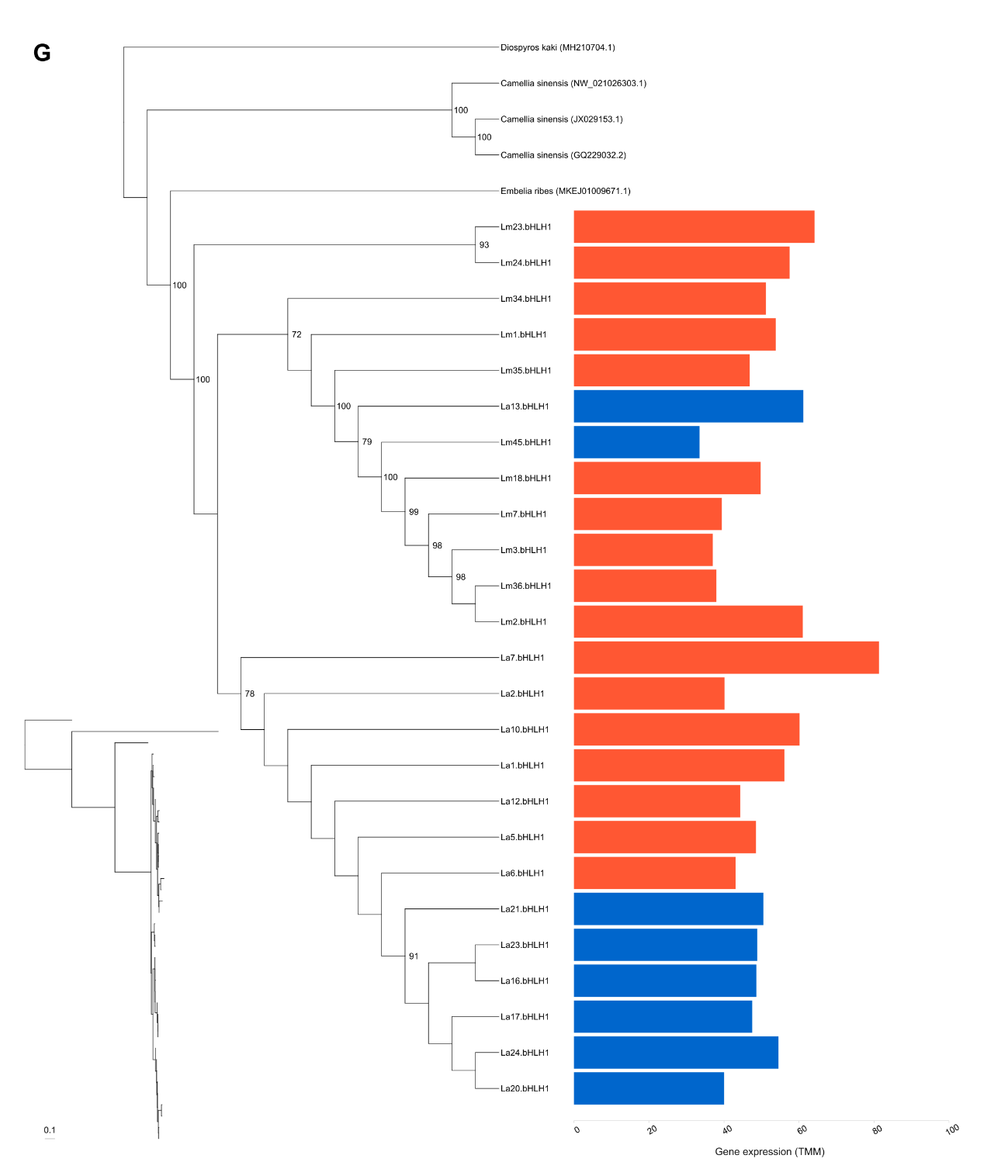


*bHLH2-2*


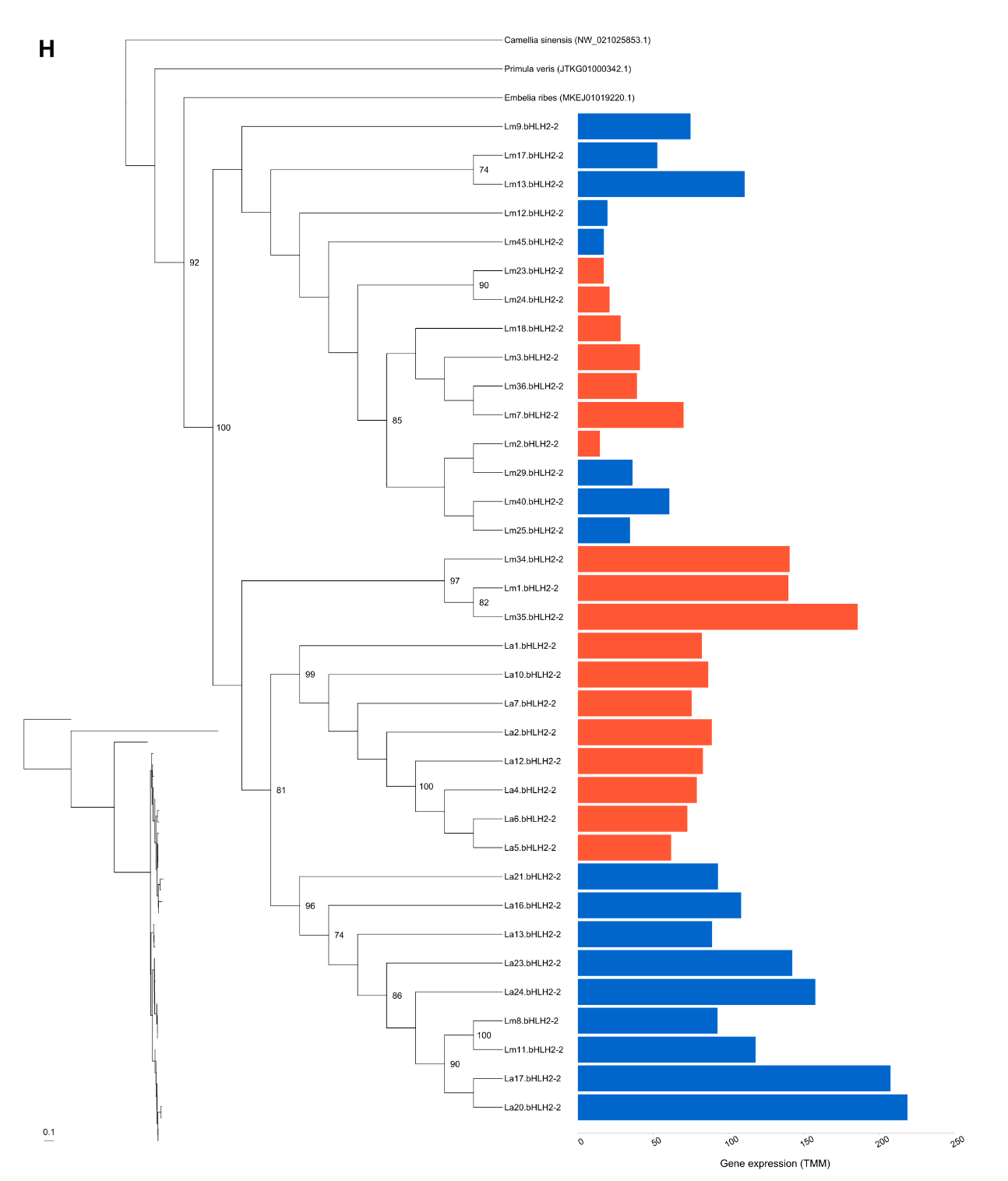


*bHLH2-3*


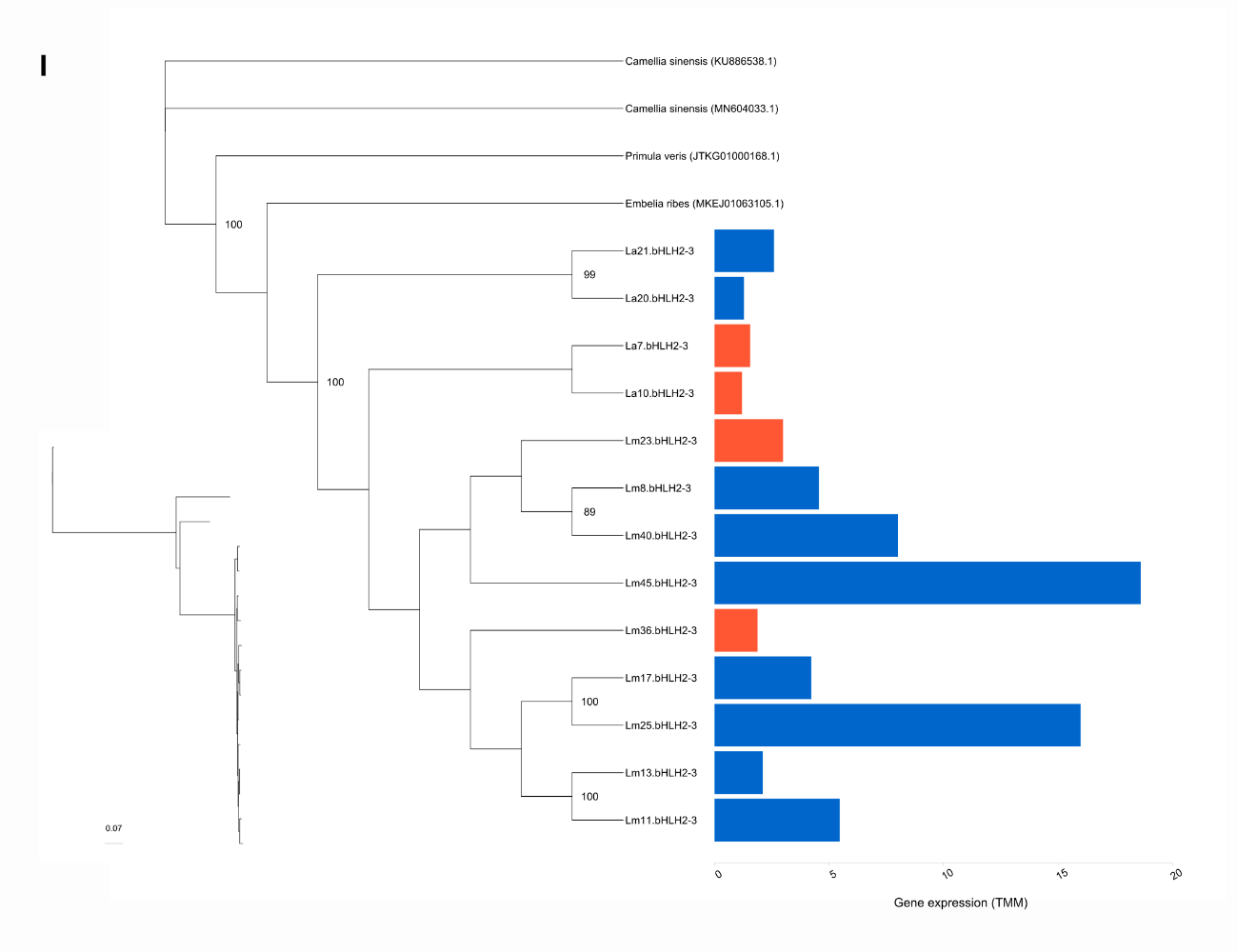


*bHLH12*


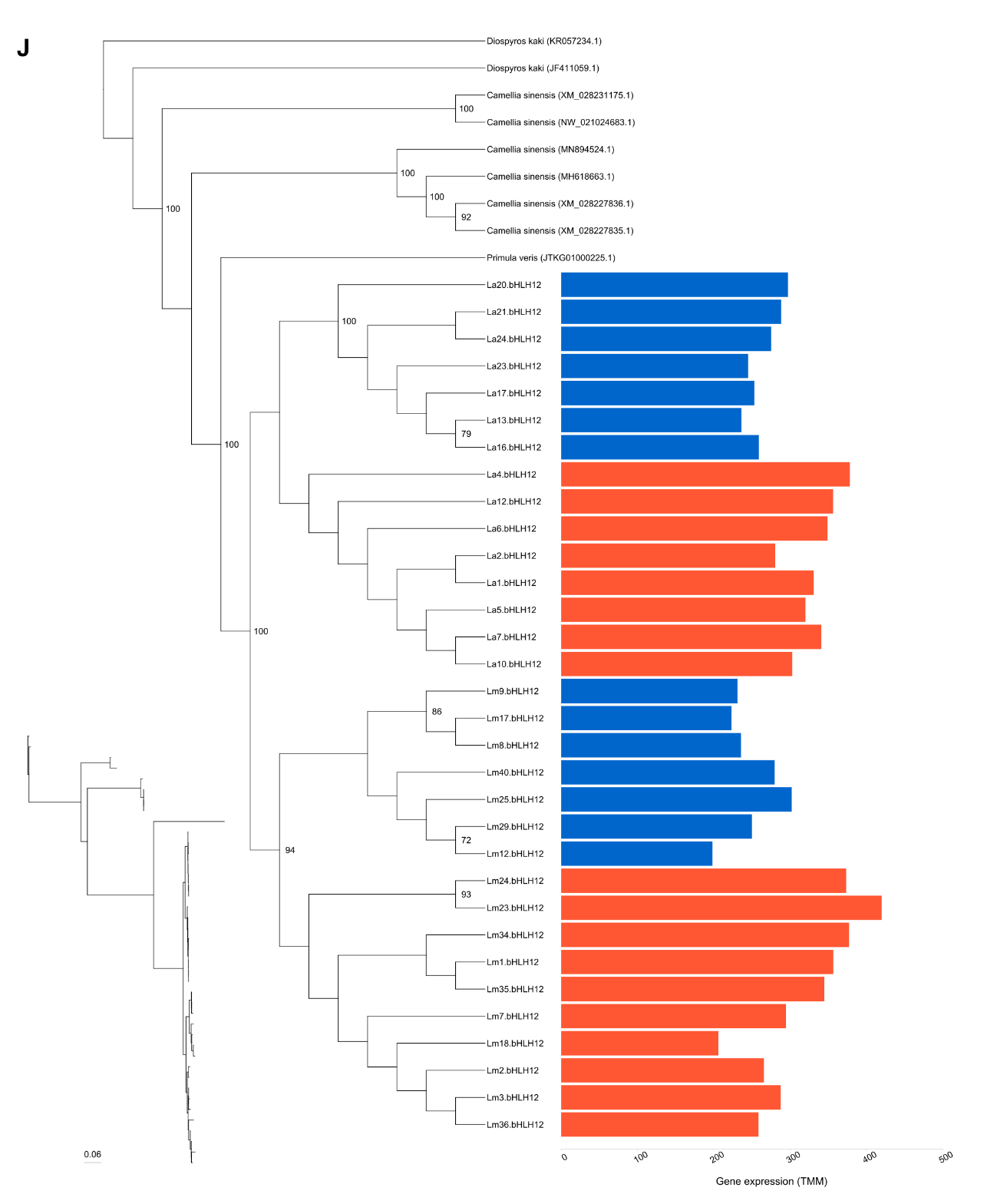


*COP1*


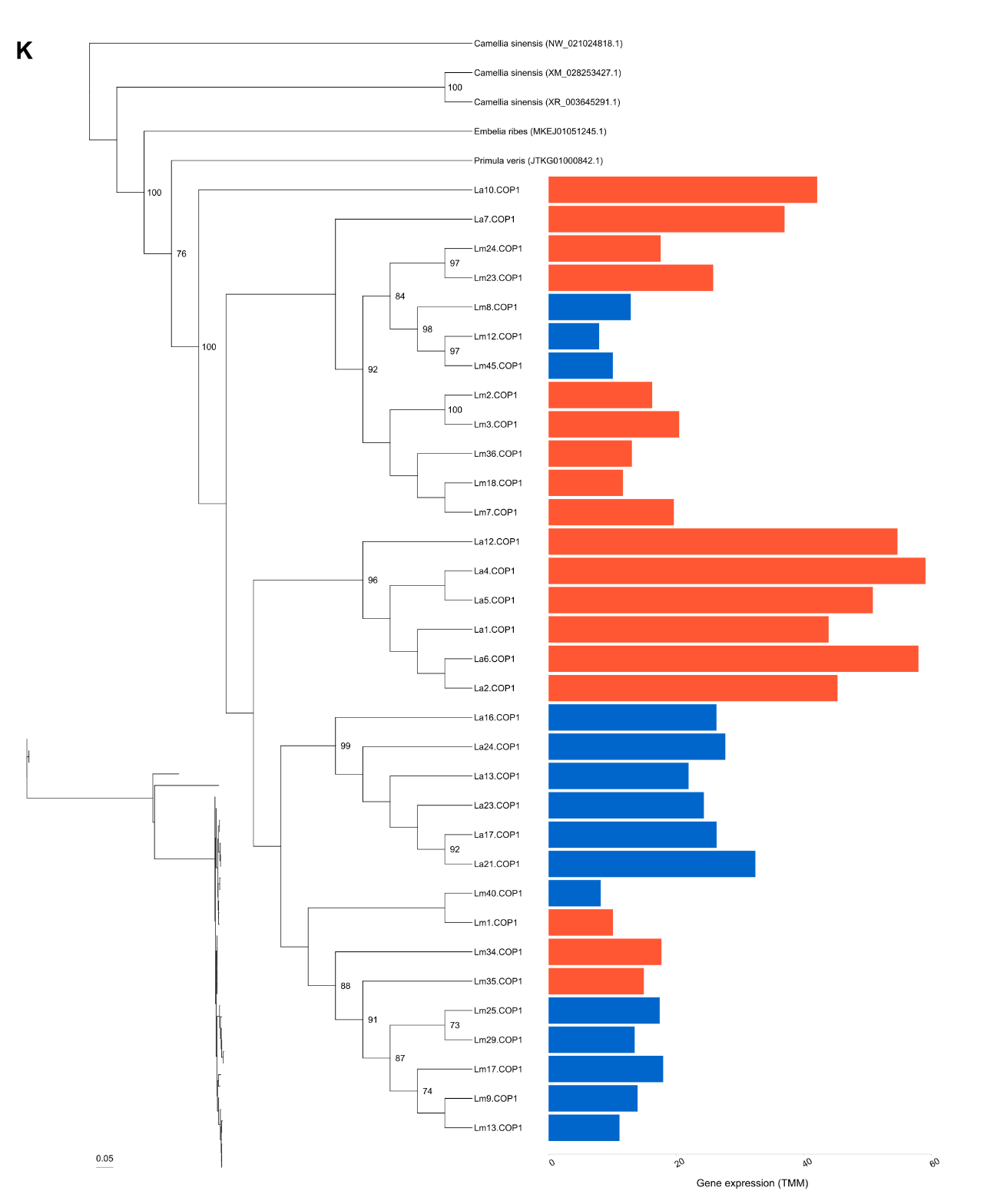


*MYB4-1*


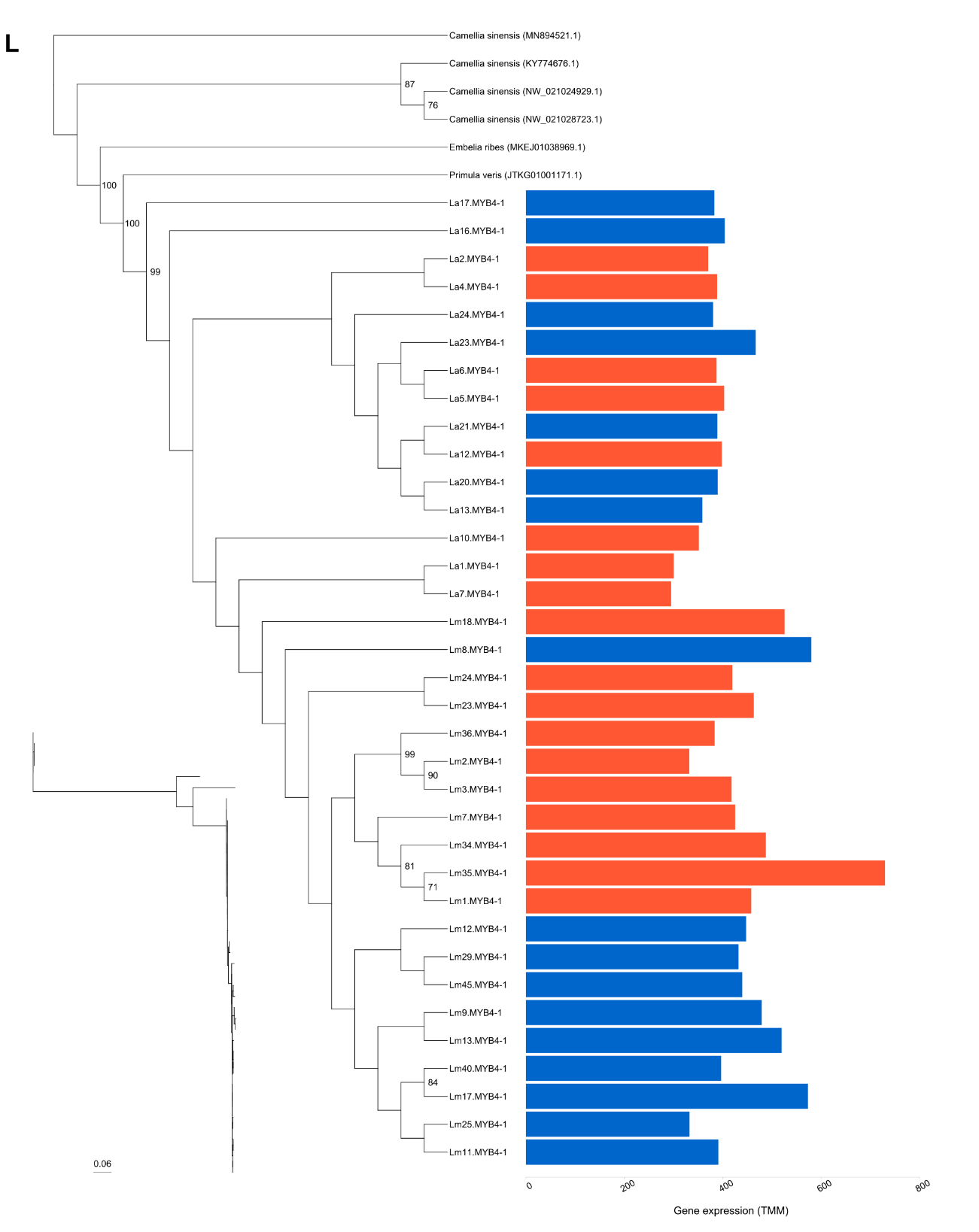


*MYB4-2*


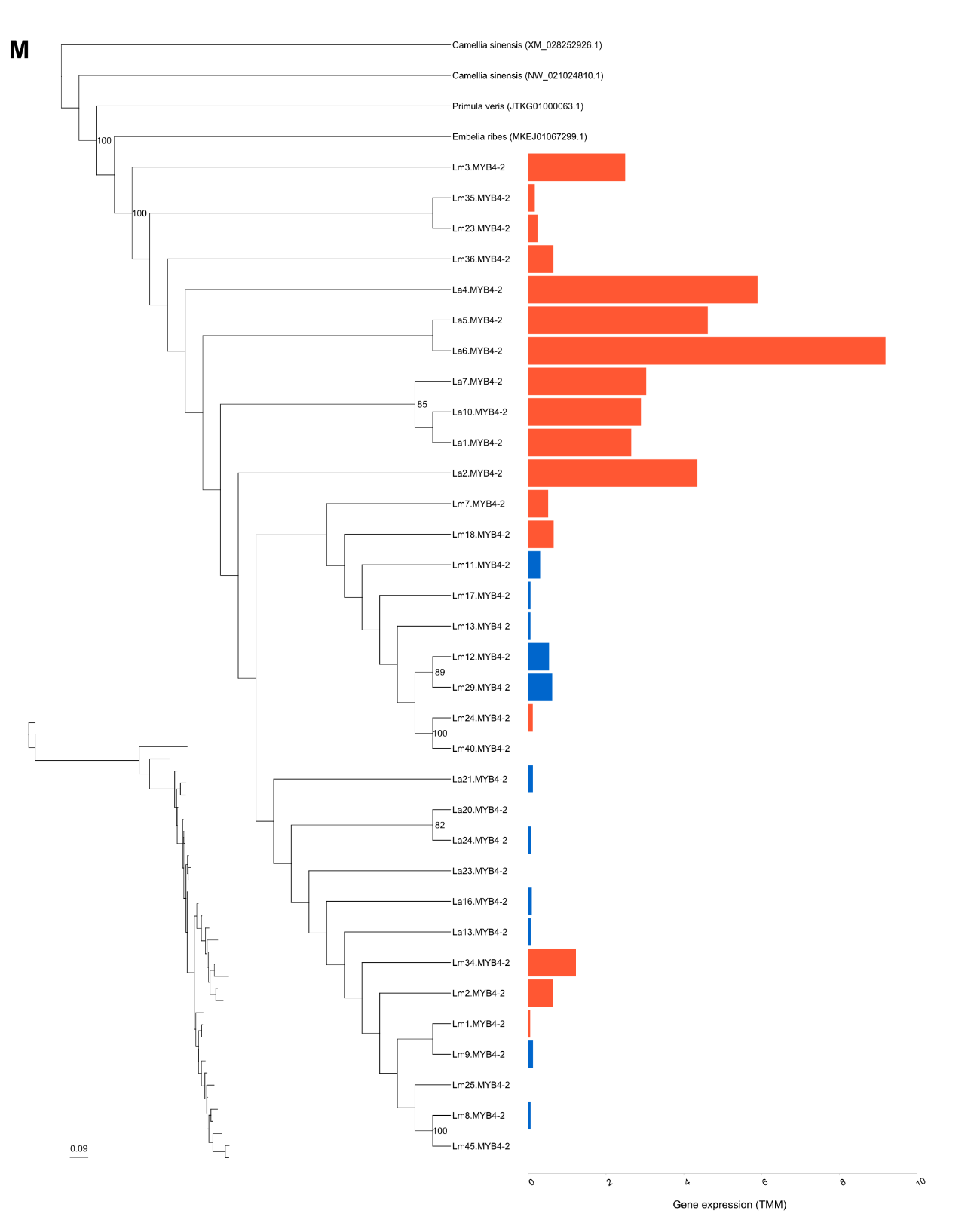


*MYB16-1*


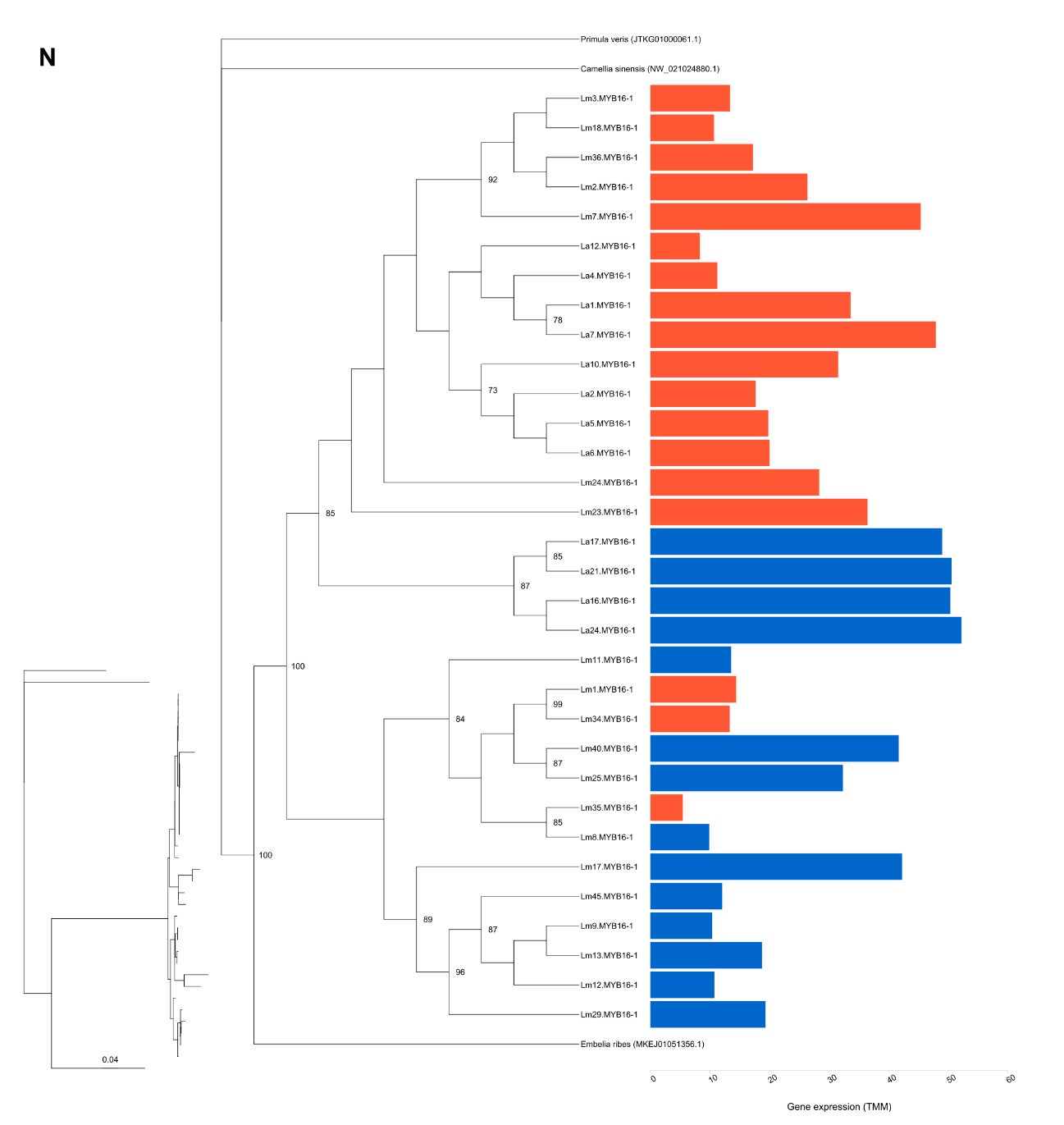


*MYB16-2*


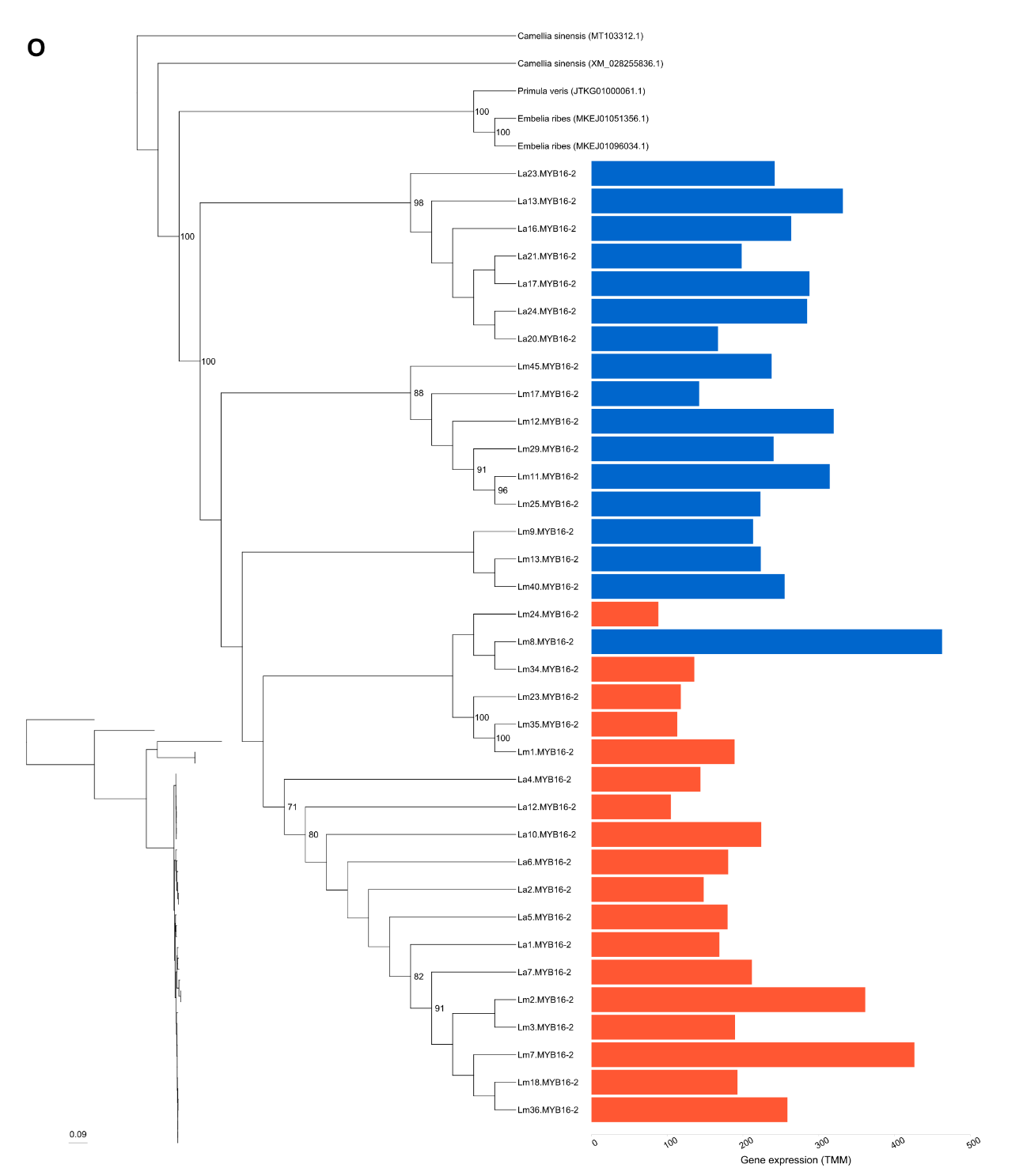


*SPA1*


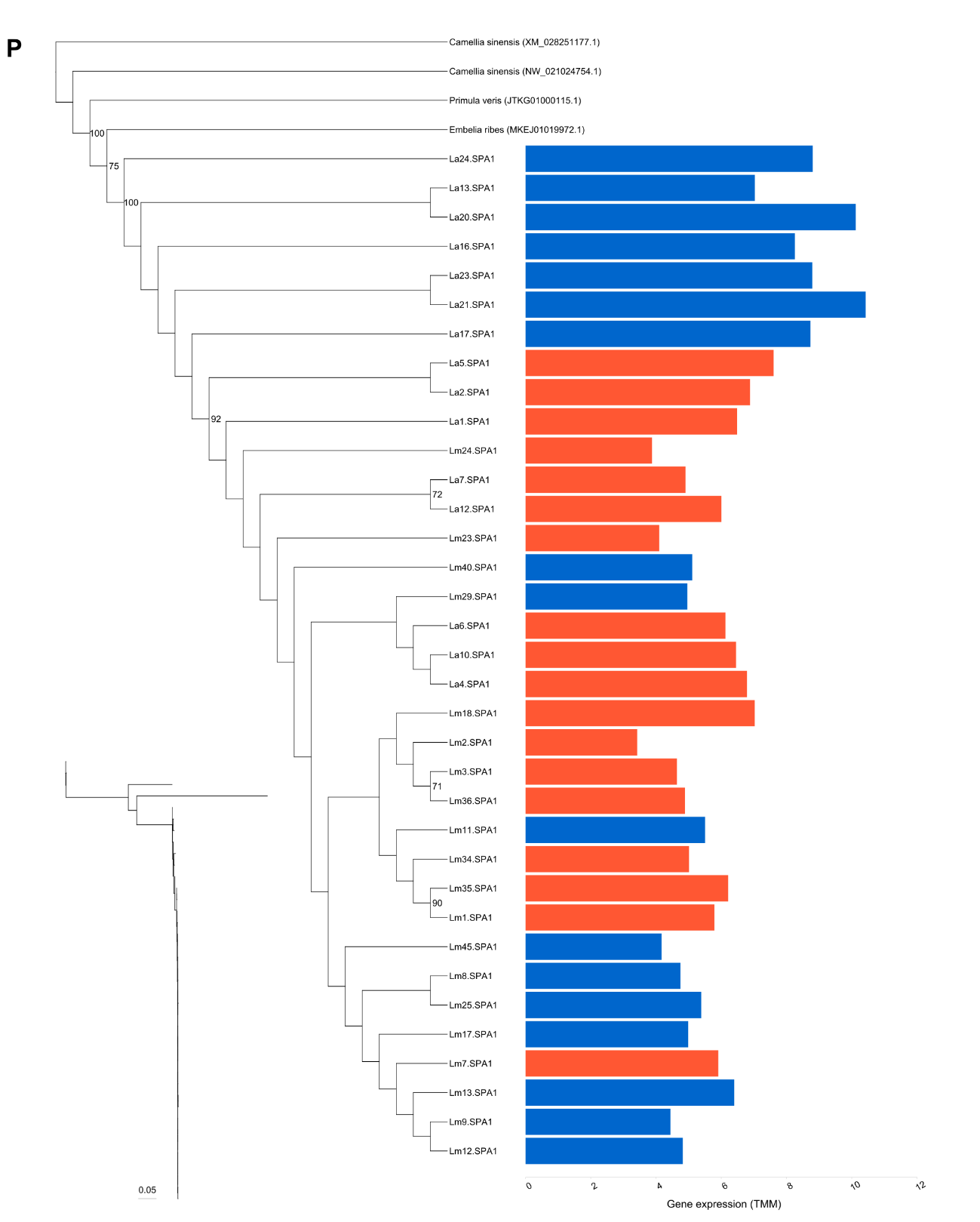


*TTG1*


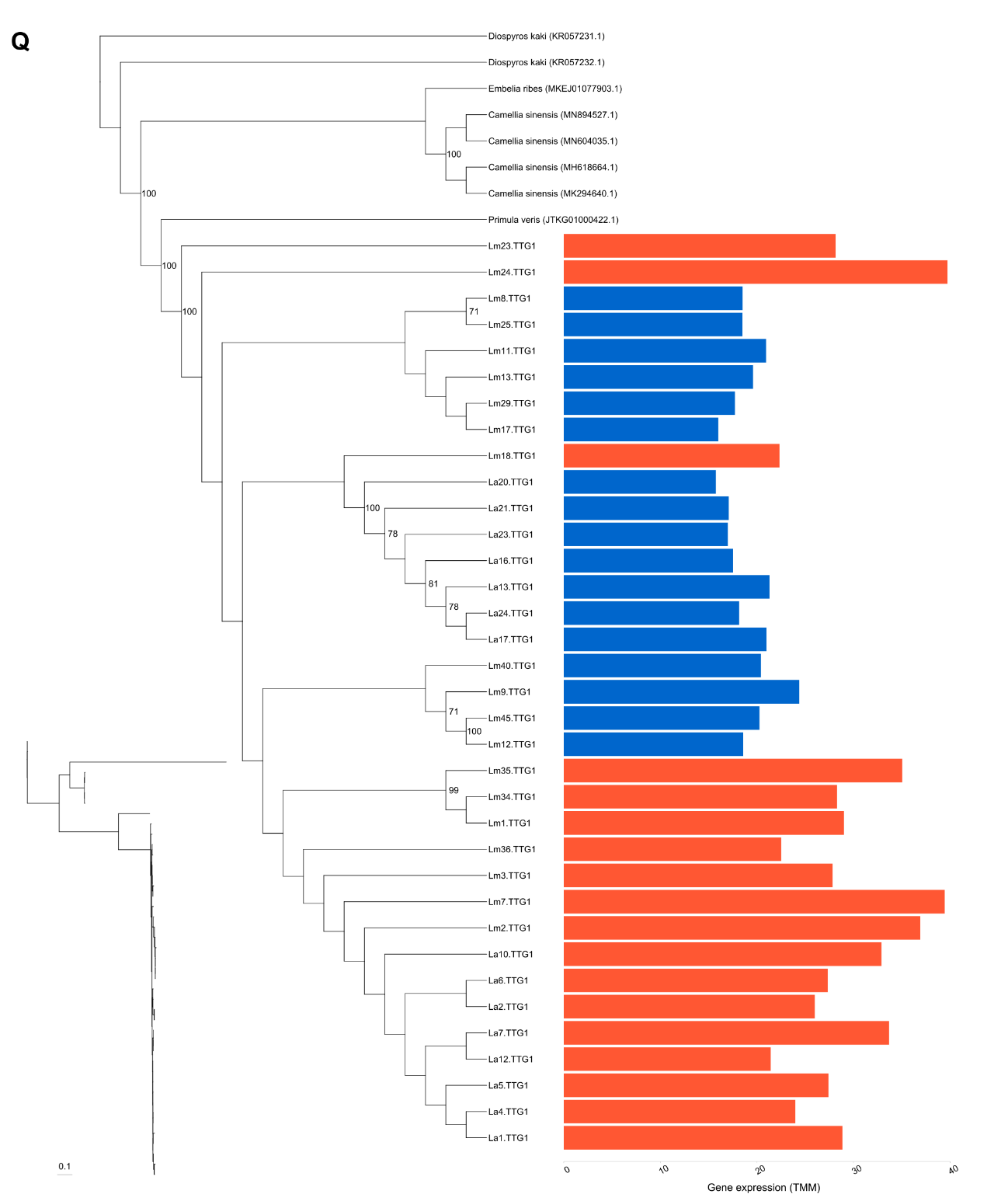


**Supplementary Figure S2.** Maximum likelihood phylogenetic analysis of the coding sequence for eight structural (A-E) and 12 regulatory (F-Q) loci from blue and orange petals of *L. arvensis* and *L. monelli*. (A) *BZ1-2* and *-3*. (B) *Caffeoyl CoA-1*,*-2* and *-3*. (C) *CHS*. (D) *F3’5’H*. (E) *F3’H*. (F) *AN11a*. (G) *bHLH1*. (H) *bHLH2-2*. (I) *bHLH2-3*. (J) *bHLH12*. (K) *COP1*. (L) *MYB4-1*. (M) *MYB4-2*. (N) *MYB16-1*. (O) *MYB16-2*. (P) *SPA1*. (Q) *TTG1*. Outgroups were included when available from the genomes of closely related species (*Camelia sinensis, Embelia ribes* and *Primula veris*). Bootstrap values above 70% are provided to the right of the nodes. Inset phylograms are provided for branch length comparisons with samples in same order as the larger cladogram and contain a scale bar in substitutions per site. The bar plots show the gene expression level (TMM values) of blue and orange flowers.


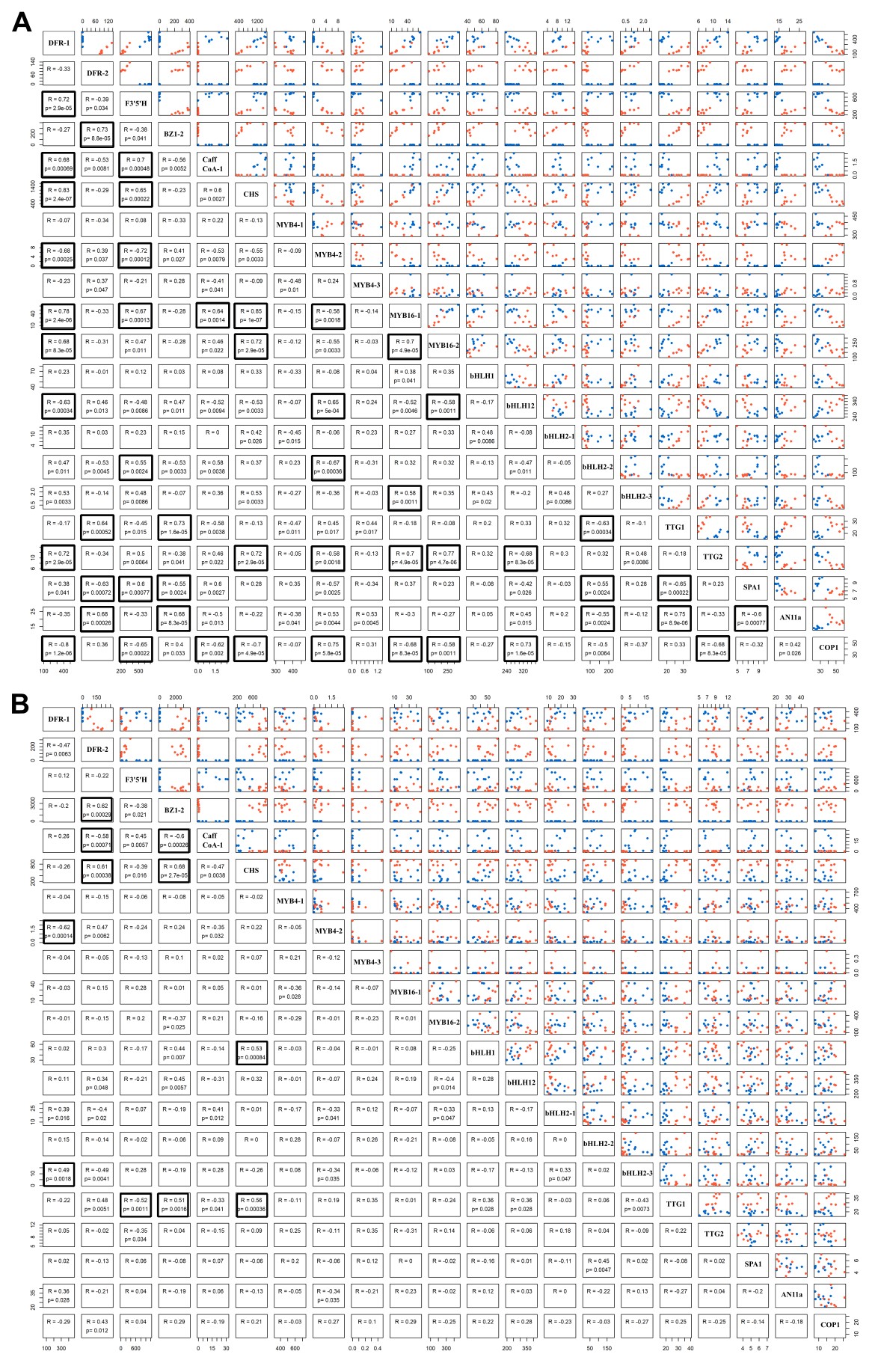


**Supplementary Figure S3.** Kendall correlations of the expression level (TMM values) between the most relevant differential expressed genes present in blue and orange *L. arvensis* (A) and *L. monelli* (B). Structural genes are *DFR-1*, *DFR-2*, *F3’5’H*, *BZ1-2*, *CaffCoA-1*, *CHS*. Regulatory genes are *MYB4-1*, *MYB4-2*, *MYB16-1*, *MYB16-2*, *bHLH1*, *bHLH12*, *bHLH2-1*, *bHLH2-2*, *bHLH2-3*, *TTG1*, *TTG2*, *SPA1*, *AN11a*, *COP1*. Correlation coefficients (R) and P values are listed below the diagonal and the raw data above the diagonal. Black boxes indicate those correlations that are significant following a Bonferroni correction. Gene names in bold are indicated in the diagonal.


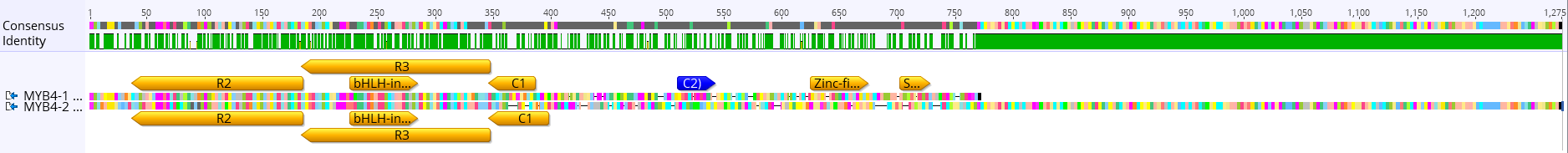


*****

*****

**Supplementary figure S4.** Alignment of the consensus sequences of *MYB4-1* and *MYB4-2* genes of *L. arvensis* and *L. monelli*. Boxes indicate the different motifs identified in the sequences: R2-R3 motif, *bHLH*-interacting motif, C1 motif, C2 motif, Zinc-finger and SID motif. Blue box locates the EAR motif (C2) for suppression function present in *MYB4-1*. * indicates the non-synonymous SNPs color differentiating position on *MYB4-2* sequence.


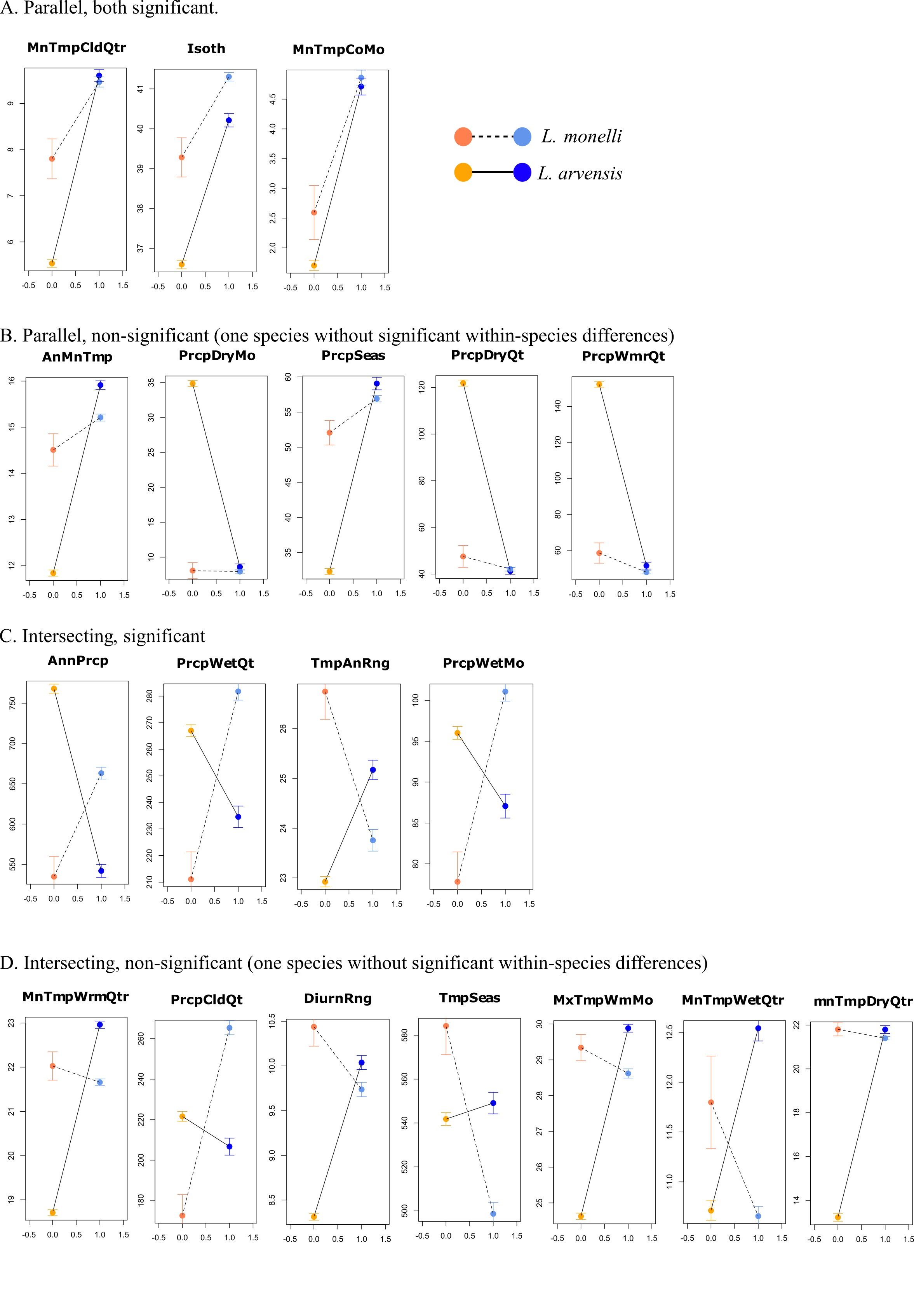
 **Supplementary Figure S5.** Climate niche modeling results for blue and orange morphs of *L. arvensis* and *L. monelli.* The 19 variables are grouped into 4 categories. (A) Parallel significant: slopes are similar (both + or both -), with orange/blue *L. monelli* significantly different AND orange/blue *L. arvensis* significantly different. (B) Parallel non-significant: slopes are similar, one or both within-species comparison is non-significant. (C) Intersecting significant: slopes are different (one +, one -), within-species differences are significant. (D) Intersecting non-significant: Slopes are different, one within-species comparisons is non-significant.


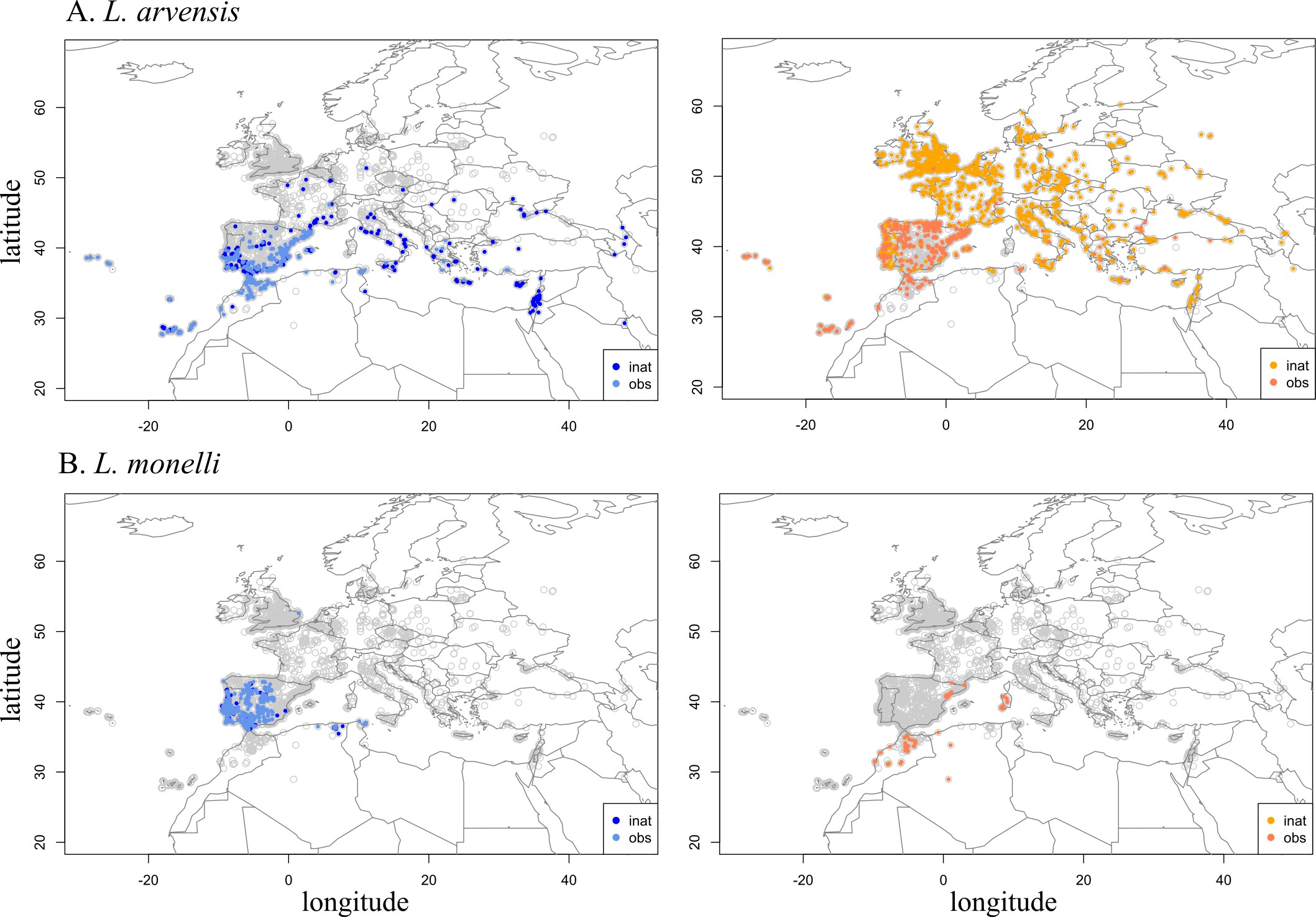


**Supplementary Figure S6.** Occurrence data used in climate niche model from individually reviewed iNaturalist records and other sources (herbarium records, personal observations). For *L. arvensis*, there are 1941 orange occurrences and 641 blue (A). For *L. monelli*, there are 662 blues and 65 oranges.

**Supplementary Table S1.** Putative flavonoid identifications of blue and orange petal extracts of *L. monelli* from the UHPLC–MS biochemical analysis (MS analysis was acquired in positive mode). Flavonoids <1% (trace) are not shown.

| Petal color | Flavonoid class | Putative flavonoid identification | N samples** | *t_R_* (min) | *λ_max_* (nm) | Mass (m/z) | Relative content (%)*** |
| --- | --- | --- | --- | --- | --- | --- | --- |
| Blue  (**n =** 10 plants) | Anthocyanin | Malvidin 3-rhamnoside | 10/10 | 5.7 | 228, 278, 526 | 476.87 | 94.4 |
|  | Anthocyanin | Delphinidin | 10/10 | 2.1 | 229, 300, 521 | variable | 1.1 |
|  | Flavonol | Kaempferol 3-glucoside / Quercetin 3-rhamnoside / Isorhamnetin 3-pentoside / Luteolin 7-glucoside | 5/10 | 7.7 | 226, 258, 291, 350 | 448.26 | 3.7 |
| Orange  (**n =** 9 plants) | Anthocyanin | Pelargonidin 3-diglucoside | 7/9 | 2.4 | 230, 277, 329, 426, 501 | 594.98 | 4.1 |
|  | Anthocyanin | Pelargonidin 3-glucoside | 9/9 | 3.1 | 231, 279, 429, 499 | 433.03 | 95.6 |

Identification was based on retention time (*t_R_*), maximum absorbance wavelengths (*λ_max_*) observed in the UV-Vis spectra of the compound that eluted at the *t_R_* indicated, and molecular mass comparisons with standards and values of previously reported flavonoids for *Lysimachia* spp. (Sánchez-Cabrera et al., 2021 and the database http://metabolomics.jp).

*The fraction of samples containing each flavonoid.

**Relative content of each putative compound in each flower color, calculated from the area under the UV-Vis peaks.

**Supplementary Table S2**. Flavonoid biosynthetic pathway genes with significant differential expression between blue- and orange-flowered *L. monelli*.

| Gene  abbreviation | Gene name | Blue expression (TMM) | Orange expression (TMM) | Log fold-change | Pathway |
| --- | --- | --- | --- | --- | --- |
| *BZ1-2* | *Anthocyanin 3-O-glucosyltransferase* | 0.31 | 2651.84 | -13.05 | ABP |
| *DFR-2* | *Dihydroflavonol 4 reductase* | 0.01 | 157.18 | -12.53 | ABP |
| *CHS* | *Chalcone synthase* | 331.90 | 784.13 | -1.24 | ABP |
| *Beta-glucosidase* | *Beta-glucosidase* | 0.09 | 4.13 | -5.37 | Non-ABP |
| *Caffeoyl CoA-1* | *Caffeoyl CoA-O-methyltransferase* | 12.73 | 0.32 | 5.29 | Non-ABP |

**Supplementary Table S3.** Summary of individual phylogenetic analyses of ABP structural and regulatory genes with high bootstrap support (>70%) indicating monophyletic clades in specific groups (genus, species, color). Genes showing orange monophyly (either for *L. arvensis*, *L. monelli* or both species) are highlighted in bold.

| Genes | *Lysimachia* | La | La-O | La-B | Lm | Lm-O | Lm-B | O | B |
| --- | --- | --- | --- | --- | --- | --- | --- | --- | --- |
| Structural genes | | | | | | | | | |
| *BZ1-2*** | 100 | 98 | - | - | - | - | - | 100 | - |
| *BZ1-3* | 100 | - | - | - | - | - | - | - | - |
| *Caff. CoA-1** | 100 | 79 | - | - | 98 | - | - | - | 100 |
| *Caff. CoA-2* | 100 | - | - | - | - | - | - | - | - |
| *Caff. CoA-3* | 100 | 100 | 99 | 98 | 85 | - | - | - | - |
| *CHS* | 100 | - | - | - | 70 | - | - | - | - |
| *DFR-1* | 100 | - | 91 | 100 | - | 92 | - | - | - |
| *DFR-2*** | 99 | - | - | - | - | - | - | 99 | - |
| *F3’5’H* | 100 | - | 98 | 86 | 93 | - | - | - | - |
| *F3’H* | 100 | 100 | 93 | 99 | - | - | 83 | - | - |
| Regulatory genes | | | | | | | | | |
| *AN11a* | 100 | - | - | - | - | - | - | - | - |
| *bHLH1* | 100 | 78 | - | 91 | - | - | - | - | - |
| *bHLH2-3* | 100 | - | - | 99 | - | - | - | - | - |
| *bHLH2-2* | 100 | - | 99 | - | - | - | - | - | - |
| *bHLH12* | 100 | 70 | - | 100 | 94 | - | - | - | - |
| *COP1* | 100 | - | - | 99 | - | - | - | - | - |
| *MYB4-1* | 99 | - | - | - | - | - | - | - | - |
| *MYB4-2* | 100 | - | - | - | - | - | - | - | - |
| *MYB16-1* | 100 |  | - | 87 | - | - | - | - | - |
| *MYB16-2* | 100 | - | - | 98 | - | - | - | - | - |
| *SPA1* | 100 | - | - | - | - | - | - | - | - |
| *TTG1* | 100 | - | - | 100 | - | - | - | - | - |

La (*L. arvensis*), Lm (*L. monelli*), O (orange), B (blue)

* Genes only present in blue petals

** Genes only present in orange petals

**Supplementary Table S4**. Fixed non-synonymous SNPs in ABP loci that differentiate color morphs and those that differentiate species.

| Gene | Position (aa) | Blue AA | Orange AA | BLOSUM62 |
| --- | --- | --- | --- | --- |
| Fixed Between Color Morphs | | | | |
| DFR-1 | 279/287^a^ | E | Q | +2 |
| MYB4-2 | 361 | M | I | +1 |
| MYB4-2 | 376 | P | S | -1 |
| Fixed Between Species | | | | |
| BZ1-3 | 435 | K/A^b^ | T | -1/0 |
| CaffCoA-3 | 36 | V | A | 0 |
| CaffCoA-3 | 200 | F | Y | +3 |
| CaffCoA-1 (only in blue)^c^ | 2 | T | A | 0 |
| CaffCoA-1 (only in blue) | 46 | E | A | -1 |
| CaffCoA-1 (only in blue) | 215 | T | A | 0 |
| CHS | 363 | R | L | -2 |
| F3'H-1 | 19 | S | Y | -2 |
| F3'H-1 | 262 | I | M | +1 |
| F3'H-1 | 324 | Q | H | 0 |
| F3'H-1 | 326 | K | E | +1 |
| F3'H-1 | 338 | A | V | 0 |
| F3'H-1 | 341 | R | L/P | -2/-2 |
| F3'5'H | 2 | E | D | +2 |
| MYB4-1 | 71 | S | T | -1 |
| MYB16-1 | 18 | T | I | +1 |

^a^ Two positions separated by a slash indicates the alignment position in *L. arvensis*/*L. monelli* (because of indels).

^b^ Two amino acids separated by a slash indicates variable in one species (BLOSUM62 values are reported with a slash in the same order).

^c^ “Only in blue” is reported when the locus was not detected in orange samples (still fixed between species, but only for blue).

**Supplementary Table S5**. Results of hand-crosses between L. arvensis and L. monelli coming from different populations.

| **Mother species** | **Father species** | **Mother plant*** | **Father**  **plant*** | **Mother color** | **Father color** | **Fruit production** |
| --- | --- | --- | --- | --- | --- | --- |
| *L. monelli* | *L. arvensis* | 630/14_1 | HIN_1.15 | Blue | Blue | 0 |
| *L. monelli* | *L. arvensis* | 630/14_2 | HIN_1.15 | Blue | Blue | 0 |
| *L. monelli* | *L. arvensis* | 630/14_3 | HIN_1.15 | Blue | Blue | 0 |
| *L. monelli* | *L. arvensis* | 630/14_4 | HIN_2.21 | Blue | Blue | 0 |
| *L. monelli* | *L. arvensis* | 630/14_5 | HIN_2.21 | Blue | Blue | 0 |
| *L. monelli* | *L. arvensis* | 630/14_1 | COR_1.6 | Blue | Orange | 0 |
| *L. monelli* | *L. arvensis* | 630/14_2 | COR_1.6 | Blue | Orange | 0 |
| *L. monelli* | *L. arvensis* | 630/14_3 | COR_1.6 | Blue | Orange | 0 |
| *L. monelli* | *L. arvensis* | 630/14_4 | COR_2.5 | Blue | Orange | 0 |
| *L. monelli* | *L. arvensis* | 630/14_5 | COR_2.5 | Blue | Orange | 0 |
| *L. monelli* | *L. arvensis* | 151/14_1 | HIN_1.15 | Blue | Blue | 0 |
| *L. monelli* | *L. arvensis* | 151/14_3 | HIN_1.15 | Blue | Blue | 0 |
| *L. monelli* | *L. arvensis* | 151/14_4 | HIN_2.21 | Blue | Blue | 0 |
| *L. monelli* | *L. arvensis* | 151/14_6 | HIN_2.21 | Blue | Blue | 0 |
| *L. monelli* | *L. arvensis* | 151/14_7 | HIN_8.7 | Blue | Blue | 0 |
| *L. monelli* | *L. arvensis* | 151/14_8 | HIN_8.7 | Blue | Blue | 0 |
| *L. monelli* | *L. arvensis* | 151/14_1 | COR_1.6 | Blue | Orange | 0 |
| *L. monelli* | *L. arvensis* | 151/14_3 | COR_1.6 | Blue | Orange | 0 |
| *L. monelli* | *L. arvensis* | 151/14_4 | COR_1.6 | Blue | Orange | 0 |
| *L. monelli* | *L. arvensis* | 151/14_6 | COR_1.52 | Blue | Orange | 0 |
| *L. monelli* | *L. arvensis* | 151/14_7 | COR_1.52 | Blue | Orange | 0 |
| *L. monelli* | *L. arvensis* | 151/14_8 | COR_1.60 | Blue | Orange | 0 |
| *L. arvensis* | *L. monelli* | HIN_1.15 | 630/14_1 | Blue | Blue | 0 |
| *L. arvensis* | *L. monelli* | HIN_2.21 | 630/14_4 | Blue | Blue | 0 |
| *L. arvensis* | *L. monelli* | HIN_2.21 | 630/14_5 | Blue | Blue | 0 |
| *L. arvensis* | *L. monelli* | COR_1.6 | 630/14_1 | Orange | Blue | 0 |
| *L. arvensis* | *L. monelli* | COR_2.5 | 630/14_5 | Orange | Blue | 0 |
| *L. arvensis* | *L. monelli* | HIN_1.15 | 151/14_1 | Blue | Blue | 0 |
| *L. arvensis* | *L. monelli* | HIN_1.15 | 151/14_3 | Blue | Blue | 0 |
| *L. arvensis* | *L. monelli* | HIN_2.21 | 151/14_4 | Blue | Blue | 0 |
| *L. arvensis* | *L. monelli* | HIN_8.7 | 151/14_8 | Blue | Blue | 0 |
| *L. arvensis* | *L. monelli* | COR_1.6 | 151/14_1 | Orange | Blue | 0 |
| *L. arvensis* | *L. monelli* | COR_1.6 | 151/14_3 | Orange | Blue | 0 |
| *L. arvensis* | *L. monelli* | COR_1.52 | 151/14_6 | Orange | Blue | 0 |
| *L. arvensis* | *L. monelli* | COR_1.60 | 151/14_8 | Orange | Blue | 0 |
| *L. monelli* | *L. arvensis* | 485/14_1 | HIN_1.15 | Orange | Blue | 0 |
| *L. monelli* | *L. arvensis* | 485/14_1 | HIN_1.15 | Orange | Blue | 0 |
| *L. monelli* | *L. arvensis* | 485/14_2 | HIN_2.21 | Orange | Blue | 0 |
| *L. monelli* | *L. arvensis* | 485/14_2 | HIN_2.21 | Orange | Blue | 0 |
| *L. monelli* | *L. arvensis* | 485/14_3 | HIN_8.7 | Orange | Blue | 0 |
| *L. monelli* | *L. arvensis* | 485/14_3 | HIN_8.7 | Orange | Blue | 0 |
| *L. monelli* | *L. arvensis* | 485/14_1 | COR_1.6 | Orange | Orange | 0 |
| *L. monelli* | *L. arvensis* | 485/14_1 | COR_1.6 | Orange | Orange | 0 |
| *L. monelli* | *L. arvensis* | 485/14_2 | COR_1.52 | Orange | Orange | 0 |
| *L. monelli* | *L. arvensis* | 485/14_2 | COR_1.52 | Orange | Orange | 0 |
| *L. monelli* | *L. arvensis* | 485/14_3 | COR_1.60 | Orange | Orange | 0 |
| *L. monelli* | *L. arvensis* | 485/14_3 | COR_1.60 | Orange | Orange | 0 |
| *L. arvensis* | *L. monelli* | HIN_1.15 | 485/14_1 | Blue | Orange | 0 |
| *L. arvensis* | *L. monelli* | HIN_2.21 | 485/14_2 | Blue | Orange | 0 |
| *L. arvensis* | *L. monelli* | HIN_8.7 | 485/14_3 | Blue | Orange | 0 |
| *L. arvensis* | *L. monelli* | COR_1.6 | 485/14_1 | Orange | Orange | 0 |
| *L. arvensis* | *L. monelli* | COR_1.52 | 485/14_2 | Orange | Orange | 0 |
| *L. arvensis* | *L. monelli* | COR_1.60 | 485/14_3 | Orange | Orange | 0 |

*Population information:

- HIN: L. arvensis. Spain, Huelva, Hinojos (37º17'42.6''N, 6º58'05''W)

- COR: L. arvensis. France, Corsica, Solenazara (41°51'19.6"N, 9°21'43.7"E)

- 630/14: L. monelli. Spain. Cádiz. Barbate. Breña de Barbate (36°10'39.3"N, 5°58'56.1"W)

- 485/14: L. monelli. Spain. Tarragona. Mont-Roig (41º5'18''N, 0º56'5''E)

- 151/14: L. monelli. Portugal. Natural Reserve das Lagoa de St André (38º0'55.2''N, 8º49'11.1''W)

**Supplementary Table S6**. Climatic differences among blue and orange morphs of *Lysimachia* species. Comparisons indicate significance of differences among morphs (e.g. between the two orange morphs or between the two color morphs within *L. arvensis*) and were Bonferroni adjusted for 76 comparisons (nineteen variables x four morph comparisons).

|  | Variable means (SE) | | | | Morph Comparisons (Kruskal Wallis p values) | | | |
| --- | --- | --- | --- | --- | --- | --- | --- | --- |
|  | arvensis orange | arvensis blue | monelli orange | monelli blue | arvensis difference | orange difference | blue difference | monelli difference |
| AnMnTmp | 11.839 (0.067) | 15.912 (0.093) | 14.508 (0.35) | 15.21 (0.076) | < 0.001 | < 0.001 | < 0.001 | 1.0 |
| MnTmpWrmQtr | 18.71 (0.07) | 22.96 (0.083) | 22.027 (0.32) | 21.659 (0.077) | < 0.001 | < 0.001 | < 0.001 | 1.0 |
| MnTmpCldQtr | 5.534 (0.084) | 9.604 (0.129) | 7.8 (0.432) | 9.464 (0.11) | < 0.001 | < 0.001 | 1.0 | 0.04 |
| AnnPrcp | 768.135 (5.65) | 541.901 (8.085) | 534.675 (25.145) | 663.231 (7.37) | < 0.001 | < 0.001 | < 0.001 | 0.002 |
| PrcpWetMo | 96.007 (0.794) | 87.062 (1.458) | 77.805 (3.642) | 101.085 (1.169) | < 0.001 | 0.003 | < 0.001 | < 0.001 |
| PrcpDryMo | 34.857 (0.427) | 8.609 (0.441) | 8.078 (1.151) | 7.946 (0.243) | < 0.001 | < 0.001 | 1.0 | 1.0 |
| PrcpSeas | 32.321 (0.425) | 59.064 (0.891) | 52.067 (1.748) | 56.902 (0.445) | < 0.001 | < 0.001 | 1.0 | 1.0 |
| PrcpWetQt | 266.99 (2.216) | 234.572 (4.038) | 211.078 (10.287) | 281.789 (3.318) | < 0.001 | < 0.001 | < 0.001 | < 0.001 |
| PrcpDryQt | 121.847 (1.295) | 41.244 (1.606) | 47.494 (4.703) | 42.091 (0.853) | < 0.001 | < 0.001 | 1.0 | 1.0 |
| PrcpWmrQt | 152.319 (1.603) | 51.462 (1.897) | 58.494 (5.645) | 47.816 (0.897) | < 0.001 | < 0.001 | 1.0 | 1.0 |
| PrcpCldQt | 221.595 (2.408) | 206.694 (4.206) | 172.61 (10.381) | 265.361 (3.44) | 0.582 | 0.022 | < 0.001 | < 0.001 |
| DiurnRng | 8.312 (0.038) | 10.038 (0.077) | 10.438 (0.217) | 9.737 (0.08) | < 0.001 | < 0.001 | 1.0 | 0.698 |
| Isoth | 36.593 (0.107) | 40.213 (0.167) | 39.281 (0.491) | 41.305 (0.108) | < 0.001 | < 0.001 | 0.002 | 0.05 |
| TmpSeas | 541.797 (2.946) | 549.092 (4.9) | 584.242 (13.062) | 498.631 (5.108) | 1.0 | 1.0 | < 0.001 | < 0.001 |
| MxTmpWmMo | 24.626 (0.087) | 29.884 (0.11) | 29.34 (0.366) | 28.619 (0.13) | < 0.001 | < 0.001 | < 0.001 | 1.0 |
| MnTmpCoMo | 1.703 (0.081) | 4.712 (0.142) | 2.594 (0.454) | 4.862 (0.126) | < 0.001 | 1.0 | 1.0 | < 0.001 |
| TmpAnRng | 22.923 (0.104) | 25.172 (0.195) | 26.747 (0.56) | 23.757 (0.219) | < 0.001 | < 0.001 | < 0.001 | < 0.001 |
| MnTmpWetQtr | 10.711 (0.098) | 12.542 (0.128) | 11.798 (0.465) | 10.655 (0.097) | < 0.001 | 1.0 | < 0.001 | 1.0 |
| mnTmpDryQtr | 13.224 (0.178) | 21.795 (0.179) | 21.802 (0.297) | 21.399 (0.08) | < 0.001 | < 0.001 | 1.0 | 1.0 |

**Supplementary Table S7**. Logistic regression models for each species on a subset on non-correlated climate variables. The best models using AIC for *L. arvensis* (Arv) and *L. monelli* (Mon) are presented. “Good models” have lower, but close AIC values and reinforce the importance of the variables included in the best model.

***L. arvensis* Best Model**

| Coefficients | Estimate | Std. Error | z value | p |
| --- | --- | --- | --- | --- |
| Intercept | 14.5510859 | 0.8911108 | 16.33 | < 0.0001 |
| PrcpSeas | -0.0453151 | 0.0028234 | -16.05 | < 0.0001 |
| Isoth | -0.1980946 | 0.0168407 | -11.76 | < 0.0001 |
| TmpSeas | -0.0070202 | 0.0005796 | -12.11 | < 0.0001 |

***L. arvensis* Good Models**

| Intercept | Isoth | MeanTemp WarmQtr | PrcpSeas | PrcWarm Mon | TmpSeas | df | Delta AIC |
| --- | --- | --- | --- | --- | --- | --- | --- |
| 47.92 | 20.240 |  | -37.950 | 2.044 | -46.670 | 5 | 1.20 |
| 58.550 | 18.270 | 1.649 | -39.18 |  | -57.010 | 5 | 1.96 |
| 45.220 | 19.610 | 5.531 | -37.94 | 2.695 | -44.090 | 6 | 2.80 |

***L. monelli* Best Model**

| Coefficients | Estimate | Std. Error | z value | p |
| --- | --- | --- | --- | --- |
| Intercept | -1.568856 | 0.635342 | -2.469 | 0.0135 |
| PrcpWetMo | -0.035533 | 0.005195 | -6.840 | < 0.0001 |
| MnTmpWetQtr | 0.229809 | 0.049652 | 4.628 | < 0.0001 |

***L. monelli* Good Models**

| Intercept | Isoth | MeanTemp WarmQtr | PrcpSeas | PrcWarm Mon | TmpSeas | df | Delta AIC |
| --- | --- | --- | --- | --- | --- | --- | --- |
| 22.280 | -81.070 | 215.70 | 20.42 | -24.93 | -20.80 | 6 | 1.68 |
| 46.480 | -89.320 | 210.80 |  | -24.22 | -44.92 | 5 | 5.67 |
| -0.02067 | -70.320 | 210.30 |  | -17.75 |  | 4 | 5.82 |

**Supplementary Table S8**. Significance values from randomization tests. They indicate whether orange or blue populations are more different from one another climatically than expected by chance.

|  | Orange divergent | Blue divergent | Conclusion |
| --- | --- | --- | --- |
| AnMnTmp | < 0.001 | 0.999 | Orange more divergent |
| MnTmpWrmQtr | < 0.001 | 0.999 | Orange more divergent |
| MnTmpCldQtr | < 0.001 | 0.999 | Orange more divergent |
| AnnPrcp | < 0.001 | 0.999 | Orange more divergent |
| PrcpWetMo | 0.205 | 0.794 | Not Significant |
| PrcpDryMo | < 0.001 | 0.999 | Orange more divergent |
| PrcpSeas | < 0.001 | 0.999 | Orange more divergent |
| PrcpWetQt | 0.039 | 0.96 | Orange more divergent |
| PrcpDryQt | < 0.001 | 0.999 | Orange more divergent |
| PrcpWmrQt | < 0.001 | 0.999 | Orange more divergent |
| PrcpCldQt | 0.796 | 0.203 | Not significant |
| DiurnRng | < 0.001 | 0.999 | Orange more divergent |
| Isoth | < 0.001 | 0.999 | Orange more divergent |
| TmpSeas | 0.702 | 0.297 | Not significant |
| MxTmpWmMo | < 0.001 | 0.999 | Orange more divergent |
| MnTmpCoMo | 0.410 | 0.958 | Not significant |
| TmpAnRng | < 0.001 | 0.999 | Orange more divergent |
| MnTmpWetQtr | 0.999 | < 0.001 | Blue more divergent |
| MnTmpDryQtr | < 0.001 | 0.999 | Orange more divergent |

**Supplementary Table S9.** Evidence for the four origin hypotheses of the flower color polymorphism in *Lm* and *La*.

| Evidence | Evidence for convergent evolution (Fig. 5A) | Evidence for introgression (Fig. 5B) | Evidence for trans-specific polymorphism (Fig. 5C) | Evidence for non-monophyly of species (Fig. 5D) |
| --- | --- | --- | --- | --- |
| UV-vis spectra | In the UV, two peaks for *Lm*-O vs. one peak + a shoulder for *La*-O and in the visible wavelengths there are some small differences in the inflection points (Fig. 1B). | 7x more variation between color morphs than within colors between species (Fig. 1B, Supplementary Fig. S1). | 7x more variation between color morphs than within colors between species (Fig. 1B, Supplementary Fig. S1). | 7x more variation between color morphs than within colors between species (Fig. 1B, Supplementary Fig. S1). |
| Biochemistry | None. | Identical anthocyanins detected in both species and no significant differences in the relative concentrations for each color morph between species (Fig. 1C). | Identical anthocyanins detected in both species and no significant differences in the relative concentrations for each color morph between species (Fig. 1C). | Identical anthocyanins detected in both species and no significant differences in the relative concentrations for each color morph between species (Fig. 1C). |
| Gene expression | Compensatory downregulation of the *F3’5’H*, *DFR-1* and *BZ1-1* in orange morphs is only significant in *La* (Fig. 2A); *CHS, OMT, 3GT* and *UGT72E* are DEGs in *La*, but not *Lm* (Fig. 2A). | Significant upregulation of *DFR-2* and *BZ1-2* in orange morphs of both species (Fig. 2A). | Significant upregulation of *DFR-2* and *BZ1-2* in orange morphs of both species (Fig. 2A). | Significant upregulation of *DFR-2* and *BZ1-2* in orange morphs of both species (Fig. 2A). |
| Expression correlations | Correlation between *DFR-2* and *TTG1* is only significant in *La* after Bonferroni correction (Supplementary Fig. S3). | *DFR-2* and *BZ1-2* are strongly correlated with each other in both species and *BZ1-2* is significantly correlated with the positive regulator *TTG1* in both species; *DFR-1* is negatively correlated with *MYB4-2* in orange morphs of both species (Supplementary Fig. S3). | *DFR-2* and *BZ1-2* are strongly correlated with each other in both species and *BZ1-2* is significantly correlated with the positive regulator *TTG1* in both species; *DFR-1* is negatively correlated with *MYB4-2* in orange morphs of both species (Supplementary Fig. S3). | *DFR-2* and *BZ1-2* are strongly correlated with each other in both species and *BZ1-2* is significantly correlated with the positive regulator *TTG1* in both species; *DFR-1* is negatively correlated with *MYB4-2* in orange morphs of both species (Supplementary Fig. S3). |
| SNPs | *F3’H* has five NS SNPs in *Lm*-orange and only one in *La*-orange (Supplementary Table S4). | *DFR-2* of orange morphs of both species have the same 13 NS SNPs which overlap with a known pelargonidin-specific domain (Fig. 2B). *MYB4-2* has two NS SNPs fixed between color morphs in both species. (Supplementary Table S4). | *DFR-2* of orange morphs of both species have the same 13 NS SNPs which overlap with a known pelargonidin-specific domain (Fig. 2B). *MYB4-2* has two NS SNPs fixed between color morphs in both species. (Supplementary Table S4). | *DFR-2* of orange morphs of both species have the same 13 NS SNPs which overlap with a known pelargonidin-specific domain (Fig. 2B). *MYB4-2* has two NS SNPs fixed between color morphs in both species. (Supplementary Table S4). |
| 22 ABP genes phylogeny | Monophyly of species (Fig. 4). | None. | Monophyly of species (Fig. 4). | None. |
| Climate niche modeling | Four variables (mostly precipitation-related) have opposing effects on color morphs in the two species (orange *Lm* = dry vs. orange *La* = wet; Supplementary Fig. S4C). Furthermore, two of the three temperature-related variables that have parallel effects on color morphs showed differences between oranges but not between blues. | Three temperature-related variables have parallel effects on color morphs for both species (orange = cooler than blue; Supplementary Fig. S4A). However, there are differences between oranges. | Three temperature-related variables have parallel effects on color morphs for both species (orange = cooler than blue; Supplementary Fig. S4A). | None. |
| Crosses | No fruits produced from crosses between species (regardless of the color morph), but fruit production when crossing morphs within species. | None. | No fruits produced from crosses between species (either color), but fruit production when crossing morphs within species. | None. |

**Supplementary Table S10.** Plant population locations and herbarium accession numbers for *Lysimachia monelli* samples used for RNA-seq and UHPLC-MS (flavonoid profiling) (marked with a X). Voucher specimens were deposited in the University of Seville Herbarium (SEV).

| Population | Color | Country | Location | Lat**.** | Long**.** | RNA-Seq | UHPLC-MS | **Accession #** |
| --- | --- | --- | --- | --- | --- | --- | --- | --- |
| 1/17-MS | Blue | Spain | Huelva. Almonte | 37.055472 | -6.541722 | X | X | SEV285033 |
| 2/17-MS | Blue | Spain | Huelva. Pinares de Hinojos | 37.217917 | -6.417889 | X | X | SEV287947 |
| 3/17-MS | Blue | Spain | Sevilla. Bormujos | 37.371861 | -6.082139 | X | X | SEV287606 |
| 4/17-MS | Blue | Spain | Sevilla. Bellavista | 37.330694 | -5.964528 |  | X | SEV287605 |
| 6/17-MS | Blue | Spain | Sevilla. Dos Hermanas | 37.275042 | -5.932889 | X | X | SEV286835 |
| 7/17-MS | Blue | Spain | Cádiz. Carretera entre Borneo y Espera | 36.836028 | -5.752167 |  | X | SEV286849 |
| 10/17-MS | Blue | Spain | Sevilla. Entre los Molares y Utrera. Km 5 | 37.162364 | -5.760236 | X | X | NA |
| LM_8 | Blue | Spain | Cádiz. Barbate. Breña de barbate | 36.177583 | -5.982250 | X | X | SEV286494 |
| LM_24 | Blue | Portugal | Carrapateira | 37.193333 | -8.905278 | X | X | SEV286463 |
| LM_28 | Blue | Portugal | Sainto André | 38.015333 | -8.819750 |  | X | SEV284791 |
| ES_19 | Orange | Spain | Tarragona. Urb. Sant Jordi D'Alfama | 40.924883 | 0.813133 | X |  | SEV285130 |
| SAR_2 | Orange | Italy | Sardinia. Isola de Sant Pietro, Cala Fico | 39.154967 | 8.228783 | X | X | SEV287100 |
| SAR_3 | Orange | Italy | Sardinia. Isola de Sant Pietro, Cabo Sandalo, Faro | 39.144100 | 8.224050 | X | X | SEV287102 |

| SAR_6 | Orange | Italy | Sardinia. Sa Duchesa | 39.372417 | 8.597500 | X | X | SEV287085 |
| --- | --- | --- | --- | --- | --- | --- | --- | --- |
| MAR_2 | Orange | Morocco | Carretera de El Hajeb a Azrou | 33.527778 | -5.306944 |  | X | SEV289309 |
| MAR_4 | Orange | Morocco | Rif. Chefchaouene | 35.183767 | -5.224550 |  | X | SEV289310 |
| MAR_8 | Orange | Morocco | Medio Atlas. Kenifra. Entre Boufekran y Kenifra | 33.618217 | -5.430767 | X | X | SEV289311 |
| MAR_10 | Orange | Morocco | Rif. Ouezzane | 34.880000 | -5.530000 |  | X | SEV289312 |
| MAR_12 | Orange | Morocco | Medio Atlas. Kenifra | 33.027933 | -5.324650 |  | X | SEV289314 |
| MAR_30 | Orange | Morocco | Zerhoun | 34.069083 | -5.455567 |  | X | SEV289315 |

**Supplementary Table S11.** Summary of sequencing and assembly results for the 20 *L. monelli* petal samples.

| Sample | Population | Flower color | Number of reads | Number of filtered reads | Filtered reads aligned to the assembly (%) | Number of transcripts | Number of trinity genes* |
| --- | --- | --- | --- | --- | --- | --- | --- |
| Lymo_1 | ES_19 | orange | 19,356,011 | 18,421,349 | 92.48 | 47,377 | 26,986 |
| Lymo_2 | SAR_3 | orange | 17,662,750 | 16,866,036 | 93.30 | 45,323 | 27,137 |
| Lymo_3 | SAR_2 | orange | 18,038,810 | 17,237,553 | 93.16 | 48,194 | 29,126 |
| Lymo_7 | SAR_6 | orange | 17,084,149 | 16,312,231 | 92.11 | 49,194 | 29,265 |
| Lymo_18 | SAR_6 | orange | 17,166,042 | 16,255,988 | 90.86 | 48,459 | 28,927 |
| Lymo_23 | MAR_8 | orange | 16,537,229 | 15,503,176 | 92.47 | 48,701 | 28,213 |
| Lymo_24 | MAR_8 | orange | 16,709,072 | 15,886,953 | 92.23 | 49,265 | 28,074 |
| Lymo_34 | ES_19 | orange | 17,230,125 | 16,466,487 | 92.21 | 49,477 | 28,830 |
| Lymo_35 | ES_19 | orange | 23,467,043 | 22,697,727 | 92.73 | 46,959 | 27,097 |
| Lymo_36 | SAR_2 | orange | 16,981,334 | 15,773,601 | 92.57 | 52,169 | 31,542 |
| Lymo_40 | 2/17-MS | blue | 18,359,772 | 17,602,178 | 93.03 | 46,380 | 26,228 |
| Lymo_45 | LM_8 | blue | 17,828,507 | 16,984,718 | 92.32 | 48,951 | 28,143 |
| Lymo_8 | LM_24 | blue | 16,850,071 | 16,124,259 | 91.26 | 45,973 | 26,624 |
| Lymo_9 | 1/17-MS | blue | 18,050,225 | 17,338,160 | 92.84 | 45,891 | 26,354 |
| Lymo_11 | 6/17-MS | blue | 19,073,958 | 18,182,529 | 92.42 | 47,224 | 26,815 |
| Lymo_12 | LM_8 | blue | 18,350,299 | 17,810,293 | 93.54 | 44,695 | 25,856 |
| Lymo_13 | 10/17-MS | blue | 17,255,892 | 16,498,600 | 92.63 | 46,720 | 26,591 |
| Lymo_17 | 3/17-MS | blue | 17,405,546 | 16,803,173 | 93.02 | 47,313 | 27,096 |
| Lymo_25 | 3/17-MS | blue | 16,982,762 | 15,953,718 | 92.29 | 48,271 | 27,650 |
| Lymo_29 | 6/17-MS | blue | 16,331,233 | 15,578,920 | 93.01 | 45,869 | 25,935 |

* Trinity genes were limited to those having TMM > 1
